# Genetically engineered cellular nanoparticles loaded with curcuminoids for cancer immunotherapy

**DOI:** 10.7150/thno.99033

**Published:** 2024-10-07

**Authors:** Yifang Liao, Chenchen Zhao, Yuanwei Pan, Yiming Guo, Lujie Liu, Jicheng Wu, Yunjiao Zhang, Lang Rao, Qi Li

**Affiliations:** 1 Department of Urology, The First Affiliated Hospital of Zhengzhou University, Zhengzhou 450052, China.; 2 Institute of Chemical Biology, Shenzhen Bay Laboratory, Shenzhen 518132, China.; 3 School of Medicine, South China University of Technology, Guangzhou 510006, China.; 4 Cancer Center, Renmin Hospital of Wuhan University, Wuhan 430060, China.

**Keywords:** genetic engineering, cell membrane nanovesicles, immunogenic cell death, immune checkpoint blockade, cancer immunotherapy

## Abstract

**Background:** Inducing immunogenic cell death (ICD) is a promising strategy to enhance immune responses for immune checkpoint blockade (ICB) therapy, but the lack of a simple and effective platform to integrate ICD and ICB therapy limits their clinical application.

**Methods:** Here, we developed programmed cell death protein 1 (PD1)-overexpressing genetically engineered nanovesicles (NVs)-coated curcumin (Cur)-loaded poly (lactic-co-poly-polyglycolic acid) nanoparticles (PD1@Cur-PLGA) to integrate ICD and ICB therapy for enhancing tumor immunotherapy.

**Results:** Genetically engineered NVs greatly enhanced the tumor targeting of nanoparticles, and the PD1 on NVs dramatically blocked the PD1/PDL1 signaling pathway and stimulated antitumor immune responses. Meanwhile, the delivered Cur successfully induced tumor cell apoptosis and activated ICD by inhibiting NF-κB phosphorylation and Bcl-2 protein expression and activating caspase and Bax apoptotic signaling. By synergizing the ICD effect of Cur and the PD1/PDL1 axis blocking function of genetically engineered NVs, the PD1@Cur-PLGA enhanced the intratumoral infiltration rate of mature dendritic cells and CD8^+^ T cells in tumor tissues, resulting in significantly inhibiting tumor growth in breast and prostate tumor-bearing mouse models.

**Conclusion:** This synergistic ICD and ICB therapy based on genetically engineered NVs provides a low-cost, safe, and effective strategy to enhance cancer immunotherapy.

## Introduction

Cancer remains one of the most significant public health concerns worldwide 1, 2. A more profound understanding of the complex interaction between cancer and the immune system has led to the development of numerous practical therapeutic strategies that utilize the host immune system to attack and eliminate cancer cells. These developments have significantly altered the landscape of cancer therapy 3, 4. Especially for monoclonal programmed death 1 (PD1) and PD1 ligand (PDL1) antibody-mediated immune checkpoint blockade (ICB) therapy has shown exceptional success in treating many cancers, including melanoma, lung cancer, breast cancer, and non-small cell cancer 5-9. However, the efficacy of ICB in stimulating effective antitumor immune responses is often much lower than expected, particularly in poorly immunogenic tumors lacking cytotoxic T-lymphocytes (CTLs) infiltration and adequate T-cell responses 10-12. Therefore, it is imperative to exploit a safe and robust strategy to strengthen the immunogenicity of solid tumors and promote activation and tumor infiltration of CTLs, thereby enhancing the antitumor immune response of ICB.

Immunogenic cell death (ICD) represents a promising approach to improve the immunogenicity of solid tumors and enhance the effects of ICB 13-15. Tumor cells receiving ICD can release damage-associated molecular patterns (DAMPs) and tumor-associated antigens, which promote dendritic cells (DCs) maturation and activate CTLs to trigger powerful tumor-specific immune responses 16, 17. Several preclinical studies have demonstrated that antitumor chemotherapeutic agents can induce ICD and stimulate the immune system, thereby greatly enhancing CTLs infiltration and ICB responses 18-20. However, chemotherapy has severe toxic side effects and may cause inflammatory responses induced by NF-κB activation and chemokine upregulation, leading to treatment resistance and tumor recurrence, thus limiting their effectiveness in inducing ICD to enhance ICB therapy 21-23. Recently, bioactive natural products such as curcumin (Cur) extracted from turmeric have attracted widespread attention in inducing ICD, as it can exert antitumor and antiproliferative activities by down-regulating NF-κB and activating caspases apoptosis signals 24, 25. Inhibiting NF-κB activation can reduce the expression of anti-apoptotic genes including Bcl-x and Bcl-2, thereby inhibiting tumor cell proliferation, survival, and cancer progression by inducing apoptosis 26, 27. Due to the enormous potential of Cur in inducing the ICD effect, it is necessary to design a nanoplatform to integrate Cur and ICB therapy to activate CTLs and enhance antitumor immune response.

Recent studies suggested that strategically integrating ICD inducer and ICB therapy into a single multifunctional nanomaterial system will exploit potential synergies and maximize antitumor efficacy by inducing the release of antigens and DAMPs and increasing the immune response rate to ICB 28-30. Although nanomaterials offer a promising approach for co-delivery of chemotherapeutic agents and monoclonal antibodies through encapsulation or chemical conjugation, the complex preparation process and high cost of antibodies hinder their widespread application 31-34. In addition, the limited antibody release efficiency after encapsulation, as well as the destruction of antibody structure after chemical modification further restrict the therapeutic efficiency of nanomaterials 35, 36. Genetically engineered cell membrane-derived nanovesicles (NVs) displaying specific proteins, such as PD1 or PDL1, offer several advantages over synthetic nanomaterials in synergistic ICD and ICB therapy, including stable expression of proteins and favorable biocompatibility 37-39. Moreover, PD1 overexpressed on NVs can competitively bind to the PDL1 receptor on the surface of tumor cells, thereby blocking the PD1/PDL1 signaling axis and restoring antitumor T-cell immune responses 6. In addition, genetically engineered NVs can inherit the functional properties of the source cells, such as long circulation, immune evasion ability, and tumor targeting ability, which could improve drug stability in circulation and enhance drug accumulation in the tumor site, thereby reducing side effects 40-43. Therefore, we believe that genetically engineered NVs may be an ideal platform to integrate ICD and ICB therapy and improve the efficacy of antitumor immunotherapy.

**Figure 1:**
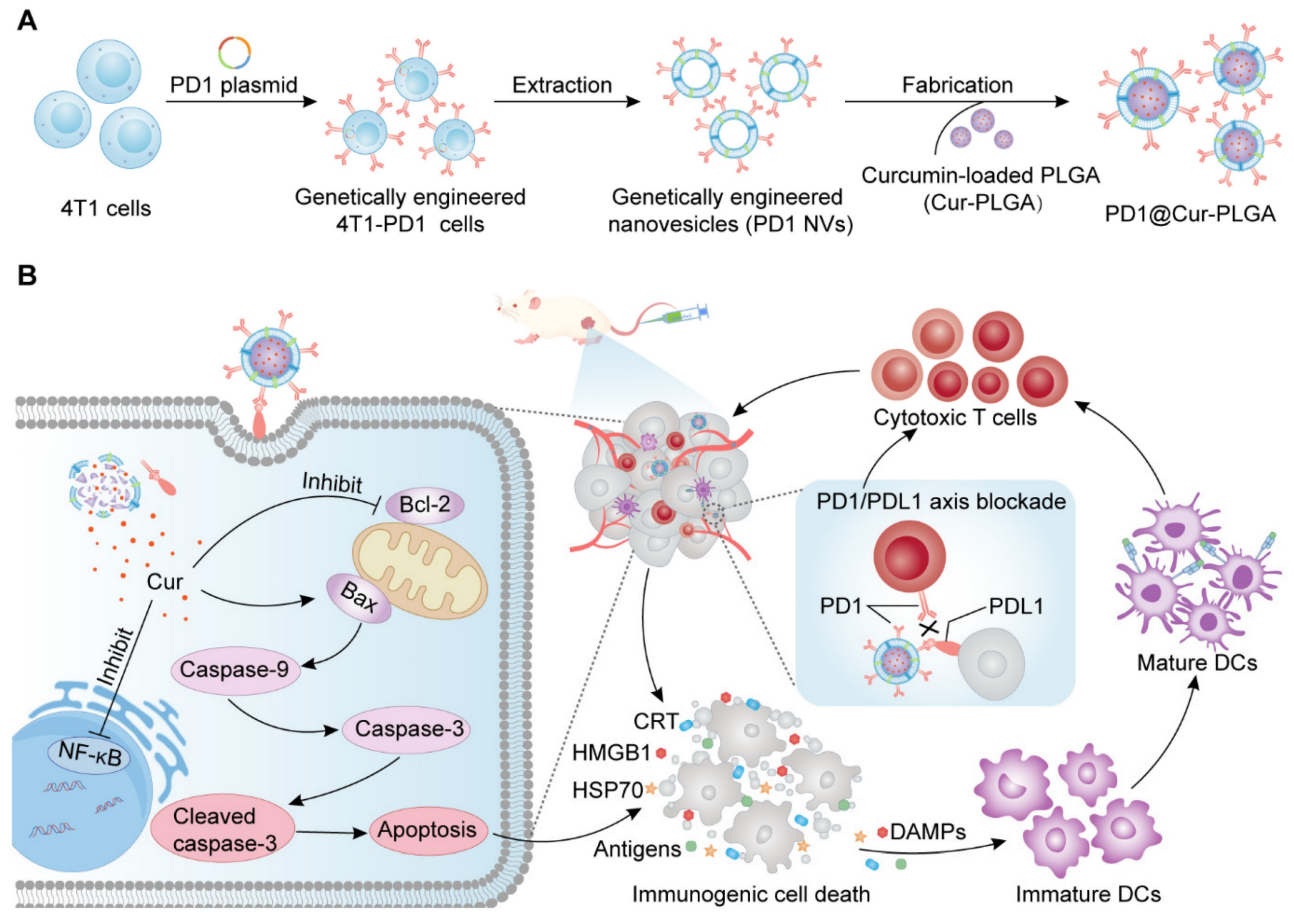
**Schematic illustration of the antitumor immune response mechanism of PD1@Cur-PLGA.** (A) The PD1@Cur-PLGA nanoparticles were prepared by coating genetically engineered NVs on the surface of the Cur-PLGA. (B) PD1@Cur-PLGA blocks PDL1 on the tumor surface through PD1/PDL1 axis, thereby improving the cytotoxicity of T cells against tumor cells. The delivered Cur efficiently induces immunogenic cell death and DAMPs release by activating caspase and Bax, promotes DCs maturation and cytotoxic T cell activation, and enhances the antitumor immune effect of PD1@Cur-PLGA.

Herein, we developed PD1-overexpressing cancer cell membrane-derived NVs-coated Cur-loaded poly (lactic-co-polyglycolic acid) (PLGA) nanoparticles (PD1@Cur-PLGA) to integrate ICD and ICB therapy and enhance antitumor immune response (Figure 1A). The NVs shell can effectively enhance tumor targeting ability, and genetically engineered PD1 can block the PD1/PDL1 signaling pathway for ICB therapy. In addition, PLGA, which has been approved by the FDA for various biomedical applications, has excellent biocompatibility and biodegradability and can improve the dispersibility of poorly water-soluble Cur. The delivered Cur can inhibit the activation of NF-κB and anti-apoptotic protein Bcl-2, further promoting the activation of caspase and Bax apoptotic signaling to induce apoptosis. Tumor antigens and DAMPs released from apoptotic cells further induce ICD and promote the maturation of DCs, thereby activating antitumor immune response of CTLs and facilitating the therapeutic effect of ICB (Figure 1B). This synergistic ICD and ICB therapy showed enhanced immune activation and favorable tumor growth inhibition in breast and prostate cancer, and provided more insights into the development of low-cost and effective tumor immunotherapy strategies.

## Materials and Methods

### Materials

Cur (Cas: 458-37-7) and PLGA (Cas: 26780-50-7) were acquired from Macklin. Wheat germ agglutinin (WGA)-conjugated Alexa Fluor 647 (WGA-AF 647, Cat: W32466) was purchased from Thermo Fisher. Anti-mouse IgG HRP antibodies (Cat: BL001A) were acquired from Biosharp. GAPDH (Cat: 5174), Anti-caspase-9 (Cat: 9508), anti-caspase-3 (Cat: 14220), and anti-cleaved caspase-3 (Cat: 9664) were acquired from Cell Signaling Technology. Other antibodies including anti-PD1 (Cat: ab211421), anti-Bax (Cat: ab32503), anti-Bcl-2 (Cat: ab182858), anti-NF-*κ*B p65 (Cat: ab32536), anti-pho-NF-*κ*B p65 (Cat: ab76302), anti-PDL1 (Cat: ab213480), donkey anti-rat IgG Alexa Fluor^®^647 (Cat: ab150075), and Goat anti-rat IgG H&L (HRP) (Cat: ab6721) were purchased from Abcam. Anti-calreticulin (CRT) antibody (Cat: GB11214), anti-high mobility group protein B1 (HMGB1) antibody (Cat: GB11103), and anti-heat shock proteins 70 (HSP70) antibody (Cat: GB15241) were acquired from ServiceBio. Enzyme-linked immunosorbent assay (ELISA) kits of HMGB1, IL-6, TNF-α, and IL-1β were purchased from Dakewe.

### Cell lines and animals

293T cells, RM-1 cells, 4T1 cells, and genetically engineered 4T1-PD1 cells were grown in a complete DMEM medium with 10% FBS and 1% penicillin-streptomycin at a cell culture incubator (Thermo Scientific, USA). Bone marrow-derived dendritic cells (BMDCs) were isolated from the bone marrow of BALB/c mice, and incubated in RPMI 1640 with 20 ng/mL mGM-CSF. BALB/c mice (female, 6-8 weeks) and C57BL/6 mice (male, 6-8 weeks) were bought from Beijing Vital River Laboratory Animal Technology Co., Ltd. All animal experiments followed the Guidelines for the Care and Use of Laboratory Animals at Shenzhen Bay Laboratory and were approved by the lab's ethics committee (Ethics No. AERL202303).

### Construction of genetically engineered 4T1-PD1 cell line

Firstly, 293T cells were transfected with plasmid (pLG0032-Plx304-PD1-mCherry) and helper plasmids (pMD2.G and psPAX2) using Lipo8000^TM^ (Beyotime) for 72 h. Then viral supernatants in the 293T cell culture medium were collected for further use. To construct PD1 overexpression-engineered 4T1-PD1 cells, 4T1 cells were cultured in six-well plates in advance. When the cell density reaches 70-80%, replace the medium with 1 mL viral supernatant containing 8 µg/mL polybrene and incubate for 8 h. The cells were supplemented with medium containing 8 µg/mL polybrene and continued to be cultured for 72 h. PD1 expression level in 4T1-PD1 cells was detected by flow cytometry (CytoFLEX LX, Beckman), confocal laser scanning microscope (CLSM) (LSM980, Zeiss), and western blot (WB) 72 h after infection.

### Preparation of genetically engineered NVs (PD1 NVs)

Genetically engineered 4T1-PD1 cells were suspended in a PBS buffer containing a mixture of phosphatase and protease inhibitors. The cells were fragmented with an ultrasonic cell disrupter, followed by centrifugation at 4,000 g for 40 min. The supernatant was collected and further centrifuged by ultracentrifuge (Coulter Optima XE-90, Beckman) at 100,000 g for 45 min, and finally the pellet was resuspended to obtain the genetically engineered cell membrane. To obtain PD1 NVs, the collected genetically engineered cell membrane was extruded sequentially through different polycarbonate membranes (800, 400, and 200 nm) using a micro-extruder (mf-1, Avestin).

### Synthesis of Cur-NVs, Cur-PLGA, and PD1@Cur-PLGA

After PD1 NVs preparation, the protein of PD1 NVs was quantified using the BCA assay (Thermo Fisher). To prepare Cur-NVs, 1 mg of PD1 NVs were mixed with 100 μg of Cur, and Cur was loaded into PD1 NVs by sonication in an ice bath for 10 min. The unencapsulated insoluble Cur was removed by centrifugation at 3,000 rpm for 10 min, and the supernatant was collected to obtain Cur-NV. Cur-PLGA was prepared through the microemulsion stirred evaporation method. Briefly, 40 mg PLGA and 4 mg Cur (*m*:*m*, 10:1) were dissolved in an organic mixed phase of dichloromethane and acetone (*v*:*v*, 4:1). The organic mixture was slowly added dropwise to the aqueous phase containing 10 mg/mL polyvinyl alcohol and stirred for 12 h. The obtained mixed emulsion was centrifuged at 20,000 g for 40 min and washed twice with deionized water, and finally, the washed sediment was resuspended with 5 mL PBS to obtain Cur-PLGA. The Cur-NVs or Cur-PLGA solution was diluted with DMSO and the absorbance of diluted Cur-NVs or Cur-PLGA at 435 nm was measured by ultraviolet spectrophotometry (UV-vis) spectroscopy (UV-2600i, Shimadzu), the loading amount of Cur was quantified by the standard curve of Cur solution. The drug loading efficiency (DLE) and drug loading capacity (DLC) were evaluated by the following formulas: DLE% = *W*/*W*_a_ × 100%; DLC = *W*/*W*_t_ × 100%. Wherein, *W* represents Cur in nanomaterials, *W*_a_ represents the amount of initial Cur loaded and *W*_t_ represents the total amount of nanomaterials after freeze-drying. Subsequently, Cur-PLGA (Cur, 1 mg/mL) and PD1 NVs (0.5 mg/mL) were mixed at a mass ratio of 2:1 (Cur-PLGA mass weight: PD1 NVs protein mass) and then sonicated in an ice bath for 30 min to obtain PD1@Cur-PLGA.

### Characterization of PD1 NVs, Cur-PLGA, and PD1@Cur-PLGA

The morphology of various nanomaterials was observed by transmission electron microscopy (TEM, JEM 1400 plus). The concentration and size distribution of various nanomaterials were detected by NS 300 (Malvern Instruments). The hydrodynamic size and zeta potential of nanomaterials, and their stability in PBS and 10% serum were measured by Zetasizer (Malvern Instruments). For fluorescence co-localization analysis, PD1 NVs were pre-labeled with DiI, Cur-PLGA were labeled with DiO, and PD1@Cur-PLGA were prepared as described previously. The fluorescence of DiI-labelled PD1 NVs and DiO-labeled Cur-PLGA was observed and pictured by CLSM, and then fluorescence co-localization analysis was performed with ImageJ software. The Cur properties of Cur-PLGA and PD1@Cur-PLGA were examined using UV-vis spectrophotometry and fluorescence spectrophotometry (RF-6000, Shimadzu), where the Cur concentration was 100 ug/mL for UV-vis analysis and 20 ug/mL for fluorescence analysis, and Cur in PBS and Cur in DMSO were used as controls. The solubility and stability of Cur, Cur-PLGA, and PD1@Cur-PLGA solutions were monitored at different times and photographed.

### *In vitro* drug release assay

The *in vitro* release of Cur was studied using the dialysis diffusion method. Briefly, Cur-PLGA or PD1@Cur-PLGA solution (1 mL) was pipetted into dialysis bags (MWCO, 6.8 kDa) and immersed in 35 mL of PBS containing 20% (*v*/*v*) Tween 80 and then kept in an incubator shaker at 37°C. At predetermined intervals, 1 ml of solution was removed and replaced with an equal volume of fresh media. The concentration of Cur in the dialysis media was determined by enzyme labeler (absorbance at 425 nm).

### Hemolysis of PD1@Cur-PLGA

Whole blood was centrifuged at 1,200 rpm to isolate red blood cells (RBCs) and washed thrice with PBS. RBCs were resuspended with 1 mL PD1 NVs, Cur-PLGA, and PD1@Cur-PLGA solution at different concentrations (0.05, 0.1, 0.2, 0.4, 0.8, and 1 mg/mL), respectively, and the mixtures were allowed to stand at 25 °C for 5 h. The samples were centrifuged at 15,000 rpm for 5 min and photographed using a digital camera. Finally, 100 μL supernatant was added to a 96-well plate and the absorption value at 570 nm was measured using an enzyme marker. The hemolysis rate was estimated based on previous studies 44, 45.

### *In vitro* cellular uptake of PD1@Cur-PLGA

Cellular uptake of PD1@Cur-PLGA was measured by flow cytometry and CLSM. For flow cytometry analysis, 4T1 cells (1 × 10^5^ cells per well) were seeded in 12-well plates and incubated with free-Cur, Cur-PLGA, and PD1@Cur-PLGA for 0, 1, 2, 4, 8, and 12 h, respectively (free-Cur and Cur-PLGA as control). After that, the cells were harvested and washed three times with PBS, then suspended in 300 μL FACs buffer and detected by flow cytometry. For fluorescence imaging analysis, 4T1 cells were seeded in confocal dishes overnight, and incubated with PD1@Cur-PLGA for 0, 1, 2, 4, 8, and 12 h. The cells were then fixed with 4% paraformaldehyde for 15 minutes and stained with DAPI for 10 minutes at room temperature and then observed with CLSM.

### CCK-8 assay

For the CCK-8 assay, 4T1 cells (1 × 10^4^ cells per well) were cultured in 96-well plates. After 12 h, the cells were treated with the cell medium containing different concentrations of PD1 NVs, Cur-PLGA, and PD1@Cur-PLGA for 24 h. After adding 10 μL CCK-8 solution to each well, the mixture was incubated for 2 h. The optical density at 450 nm was then determined under Microplate Reader (Synergy H1, Biotek) to evaluate the cell viability.

### Live/dead cell staining assay

For the live/dead cell staining analysis, 4T1 cells were incubated with Cur-PLGA and PD1@Cur-PLGA (20 μg/mL) for 24 h. Then the cells were stained with Calcein AM/PI working solution for 30 minutes, and snapped with inverted fluorescence microscopy (U-LGPS, OLYMPUS). Finally, Image J software was used to analyze the quantity of live or dead cells and calculate the cell viability.

### Cell colony formation assay

4T1 cells were seeded in 6-well plates (0.5 × 10^4^ cells per well) and cultured for four days. After treatment with Cur-PLGA and PD1@Cur-PLGA at a Cur concentration of 5 or 10 µg/mL, the cells were cultured for another three days. The colonies were photographed after being fixed with paraformaldehyde and stained with crystal violet reagent (Beyotime). Finally, the number of cell colonies was calculated using Image J software.

### TUNEL assay

Cell apoptosis was detected by TUNEL staining. Briefly, 4T1 cells were seeded in 6-well plates (3 × 10^5^ cells per well) and cultured for 12 h. After treatment with PBS, Cur-PLGA, and PD1@Cur-PLGA (20 µg/mL) for 24 h, cells were harvested and stained with the TUNEL apoptosis detection kit (YEASEN) according to the manufacturer's protocol. In short, cells were fixed in PBS containing 1% formaldehyde solution for 20 minutes at 4 °C, washed twice with cold PBS, and then treated with 0.2% Triton X-100 for 5 minutes. After washing with cold PBS, cells were treated with 1× Equilibration Buffer and incubated for 5 min. The cells were centrifuged and resuspended with TdT incubation buffer, and incubated at 37 °C for 1 h. The reaction was stopped with 20 mM EDTA, and cells were collected by centrifugation and resuspended in a PBS buffer containing 0.1% Triton X-100 solution and 5 mg/mL BSA. Cell apoptosis was analyzed by measuring Alexa Fluor 640 red fluorescence using flow cytometry.

### WB analysis

WB analysis was performed according to the general protocol from Bio-rad. Briefly, 4T1 cells (3 × 10^5^ cells per well) were cultured in 6-well plates for 12 h, then replaced with fresh medium containing Cur-PLGA and PD1@Cur-PLGA (Cur, 20 ug/mL) and cultured for another 24 h. Afterward, Cells were collected, washed, resuspended in extraction reagent, and stirred for 30 minutes at 4°C. Then the lysates were centrifuged at 12,000 rpm for 10 minutes at 4 °C. The supernatant was collected and the protein concentration was measured with a BCA assay (Thermo Fisher Scientific). The extracted lysate samples were separated by 10% SDS-PAGE and transferred to the PVDF membranes. The PVDF membranes were blocked with 5% nonfat milk for 1 h and then were incubated with primary antibodies overnight at 4 ℃, including GAPDH, Caspase-9, Bcl-2, Bax, Caspase-3, Cleaved caspase-3, NF-κB/p-65, NF-κB/pho-p65. After that, PVDF membranes were washed three times and incubated with a secondary IgG-horseradish peroxidase (HRP) antibody for 1 h. Finally, the protein on PVDF membranes was analyzed by a chemiluminescence detection system (Tanon 5200 Multi).

### Assessment of extracellular HMGB1 and ATP levels

To measure extracellular HMGB1 and ATP levels, 4T1 cells (4 × 10^4^ cells per well) were grown to 60-70% in confocal dishes and then treated with Cur-PLGA and PD1@Cur-PLGA (Cur, 20 μg/mL) for 24 h. Afterward, the supernatant of the medium was collected, and extracellular HMGB1 and ATP were detected through ELISA kits (Dakovia Biotech) and ATP assay kits (S0026, Beyotime), respectively.

### *In vitro* HMGB1 release, CRT, and HSP70 expression

To assess the effects of nanoparticle-induced ICD on the tumor cells, the release of HMGB1, the expression of CRT, and HSP70 were detected by immunofluorescence assay. 4T1 cells (4 × 10^4^ cells per well) were grown to 60-70% in confocal dishes and then treated with Cur-PLGA and PD1@Cur-PLGA (Cur, 20 μg/mL) for 24 h. After aspirating the culture medium, the cells were washed three times with PBS and then fixed with 4% paraformaldehyde for 15 minutes. After the removal of paraformaldehyde, cells were washed three times with PBS and blocked with 5% BSA buffer for 1 h. The cells were then incubated with anti-CRT antibody (GB112134, 1:500), anti-HMGB1 antibody (GB11103, 1:1,000), or anti-HSP70 antibody (GB11241, 1:1,000) at 4°C overnight. After washing away unbound antibodies with PBS, the cells were incubated with Donkey anti-rabbit IgG H&L (Alexa Fluor^R^ 647) antibody (ab150075, 1:1,000) for 1 h, then stained with DAPI for 10 minutes. The cells were observed with CLSM.

### BMDCs cultivation and DCs maturation assays

BMDCs were obtained from the femurs and tibias of female BALB/C mice (6-8 weeks old). Briefly, the isolated hind legs of mice were immersed in iodophor and then the debris and muscle tissue were removed with a 70 μm nylon mesh under sterile conditions. After red blood cell lysis, all primary cells were cultivated in RPMI-1640 complete medium containing recombinant GM-CSF (20 ng/mL) for 7 days to obtain BMDCs for further experiments. To determine the maturation of ICD-stimulated DCs, 4T1 cells were pretreated with PD1 NVs, Cur-PLGA, and PD1@Cur-PLGA (protein, 20 μg/mL; Cur, 20 μg/mL) for 24 h and then co-cultured with BMDCs (1×10^6^) for 20 h. After various treatments, the cell culture medium was centrifuged at 1,600 rpm for 5 minutes. The cell supernatants were centrifuged at 1,000 g for 20 minutes, and the supernatants were collected to quantify the levels of cytokines TNF-α, IL-6, and IL-1β that were secreted by mature DCs using ELISA assay (Dakewe Biotech). Furthermore, the DCs obtained by centrifugation were re-suspended in FACS buffer (PBS containing 2% FBS) and stained with anti-CD11c-PE (557401, BD), anti-CD80-APC (560016, BD), anti-CD86-BV650 (564200, BD), and anti-MHC-II-PE-Cy7 (107630, Biolegend) for 30 minutes at 4 °C according to the manufacturer's instructions, and then measured by a flow cytometry instrument (CytoFLEX LX, Beckman).

### *In vitro* specific targeting assay

Firstly, 4T1 cells (4 × 10^4^ cells per well) were seeded in confocal dishes and incubated overnight, then treated with Cur-PLGA, 4T1@Cur-PLGA, or PD1@Cur-PLGA (Cur, 20 μg/mL) for 4 h, respectively. After that, the cell membranes were labeled with WGA-AF 647 for 10 minutes, and the nuclei were stained with Hoechst for 10 minutes. The stained cells were washed three times with PBS and then imaged using CLSM. Hoechst, AF647, and Cur were excited at 405, 639, and 488 nm, respectively. To verify the specific targeting of PDL1 by PD1@Cur-PLGA, 4T1 cells were pretreated with anti-PDL1 antibody (ab213480, 20 μg/mL) 4 h before incubation with PD1@Cur-PLGA (20 μg/mL), and then analyzed by CLSM and flow cytometry.

### *In vivo* biodistribution assay

To establish a subcutaneous breast cancer mouse model, 4T1 cells (1×10^6^ cells) were injected into the right flank of BALB/c mice (6-8 weeks old). The tumor volume (*V*) was monitored and calculated as follows: *V*= ab^2^/2, where a and b are the length and width of the tumor. When the tumor volume reached approximately 100 mm^3^, the 4T1 tumor-bearing mice were intravenously injected with DiR-labeled Cur-PLGA and PD1@Cur-PLGA with identical Cur content (100 µL, 0.8 mg/mL). The *in vivo* living imaging was performed at predetermined time points (0, 2, 4, 8, 12, 24, 36, and 48 h) using an IVIS imaging system (PerkinElmer). 48 h after injection, tumors and major organs (heart, liver, spleen, lung, and kidney) of mice were collected and imaged using the IVIS imaging system (PerkinElmer).

### *In vivo* antitumor effects

To evaluate the antitumor effect, 4T1 tumor-bearing mice were established by subcutaneously injecting 1 × 10^6^ 4T1 cells into the right flank of mice. When the tumor volume reached approximately 50-100 mm^3^, the mice were randomly divided into five groups (*n* = 5, per group) and intravenously injected with PBS, Cur (Cur was dissolved in 5% DMSO + 5 % Tween 80 + 30% PEG300 + PBS), PD1 NVs, Cur-PLGA, and PD1@Cur-PLGA on day 0, 2, 4, and 6 with a Cur equivalent dose of 4 mg/kg. The body weight and tumor growth of mice were recorded on days 0, 2, 4, 6, 8, 10, 12, 14, and 16. At the end of the treatment, all mice were euthanized. The weights and size of tumor tissues were recorded, and the major organs and blood of mice were collected for further analysis including H&E staining, and blood biochemistry assay.

### *In vivo* anti-tumor efficacy against RM-1 prostate tumor

RM-1 cells (5 × 10^5^ cells) were injected subcutaneously into the right flank of male C57BL/6 mice (6-8 weeks). When the RM-1 tumor grew to about 50-100 mm^3^, the mice were randomly divided into two groups and were intravenously injected with PBS and PD1@Cur-PLGA (Cur, 4 mg/kg) at day 0, 2, 4, 6. The tumor volume and body weight of mice were recorded every other day. At the end of treatment, mice were sacrificed and tumors were dissected for analysis of H&E, Ki67 staining, and CD8 immunofluorescent.

### *In vivo* antitumor immunity

4T1 tumor-bearing mice were divided into five groups and received the same treatment as described above. Tumor tissues were collected 24 h after the last administration and cut into small pieces, then digested with 1640 medium containing hyaluronidase (0.1 mg/mL), DNase I (25 U/mL), and collagenase IV (0.2 mg/mL) at 37 ℃ for 40 minutes. After that, the tumor samples were abraded and filtered through a 70 µm nylon cell strainer to form a single-cell suspension. The tumor single-cell suspension was centrifuged at 600 g for 10 minutes at 4 ℃, and cell precipitates were collected and re-suspended with 5 mL erythrocyte lysate and lysed for 5 minutes. After erythrocyte lysis, the cells were centrifuged at 600 g for 10 minutes at 4 ℃ and resuspended in 5 mL FACs buffer (2% FBS, 2 mM EDTA, and 1× PBS). To detect the infiltration of CD8^+^ T cells and the maturation of DCs in the tumor tissue, 1 × 10^6^ cells were stained with fluorescence-labeled antibodies Live/Death-BV510 (77143, Biolegend), anti-CD45-APC/Cyanine 7 (557659, BD), anti-CD3-BUV395 (740268, BD), anti-CD8-PerCP/Cyanine 5.5 (551162, BD), anti-CD11c-BV605 (117334, Biolegend), anti-CD80-BV421 (562611, BD), and anti-CD86-BV650 (564200, BD) according to the manufacturer's instructions. Cells were measured on a flow cytometer (BD LSRFortessa™) and the results were analyzed by FlowJo software (version 10.8.1). Immunofluorescence images of CRT, HMGB1, and PDL1 stained tumor sections were acquired using the standard protocol. Moreover, the TNF-α, IL-6, and IL-1β levels in tumor tissues were measured by ELISA assays according to the manufacturer's instructions.

### *In vivo* toxicity evaluation

To evaluate the* in vivo* toxicity of nanoparticles, major organs, and whole blood were collected from 4T1 tumor-bearing mice 48 h after administration. Moreover, after antitumor treatment, major organs and whole blood of 4T1 tumor-bearing mice were also collected at the end of treatment. The liver, spleen, kidney, heart, and lung were fixed with 4% paraformaldehyde, stained with hematoxylin and eosin, and finally examined using a research slide scanner (VS200, Demo). Blood was collected without an anticoagulant and allowed to clot for 10 min at room temperature, then serum was separated by centrifugation at 3,000 rpm to evaluate serum chemical parameters including alanine aminotransferase (ALT), aspartate aminotransferase (AST), alkaline phosphatase (ALP), creatinine (CREA), urea (UREA), and urine acid (UA).

### Statistics analysis

All data were analyzed as the mean ± standard deviation (S.D.) obtained from at least three independent trials and plotted with Prism version 8.0 software (GraphPad). Statistical differences were determined by one-way analysis of variance (ANOVA) and Tukey's test, and where appropriate, two-way ANOVA and Tukey's test were performed using multiple comparisons. *P* < 0.05 was considered statistically significant.

## Results and Discussion

### Preparation and characterization of PD1@Cur-PLGA

**Figure 2:**
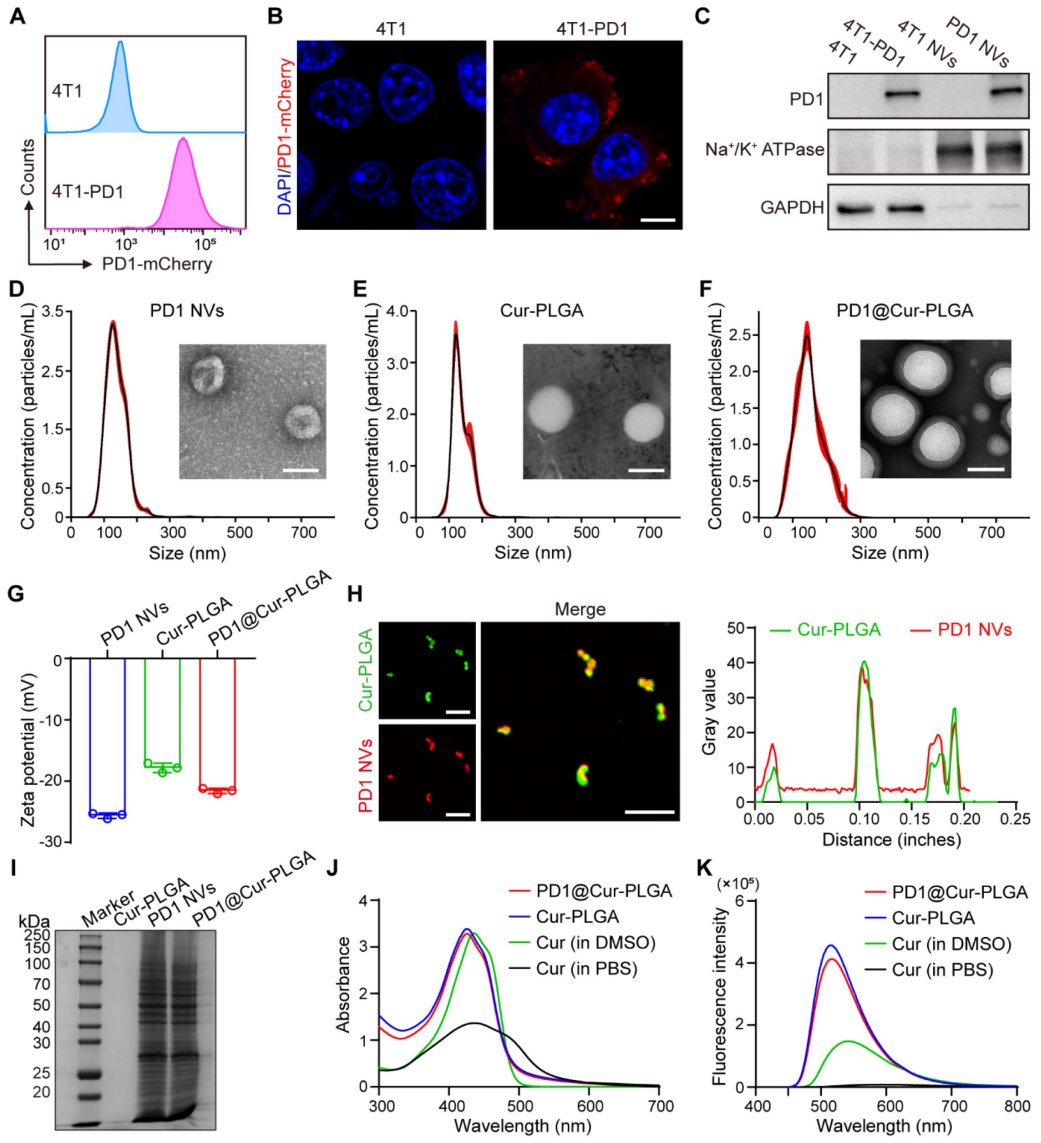
** Preparation and characterization of PD1@Cur-PLGA.** (A, B) Flow cytometry analysis (A) and immunofluorescence images (B) of PD1 expression on 4T1 and 4T1-PD1 cells. Scale bar, 10 μm. (C) WB analysis of PD1 expression in 4T1-PD1 cells and PD1 NVs. (D-F) Size distribution and corresponding TEM images of PD1 NVs (D), Cur-PLGA (E), and PD1@Cur-PLGA (F). Scale bar, 100 nm. (G) Zeta potential of PD1 NVs, Cur-PLGA, and PD1@Cur-PLGA. (H) Confocal fluorescence images and co-location analysis of PD1@Cur-PLGA. Scale bar, 10 μm. (I) SDS-PAGE analysis of PD1 NVs, Cur-PLGA, and PD1@Cur-PLGA. (J, K) UV-vis absorption spectrum (J) and fluorescence intensity (K) of Cur (in PBS), Cur (in DMSO), Cur-PLGA (in PBS), and PD1@Cur-PLGA (in PBS). Data are presented as mean values ± S.D (*n* = 3).

To prepare genetically engineered 4T1-PD1 cells, a lentiviral plasmid encoding the PD1 gene domain was transfected into 4T1 cells *via* lentiviral transfection methods. Flow cytometry analysis demonstrated that PD1 was successfully constructed on 4T1-PD1 cells (Figure 2A). The strong mCherry fluorescence signal of 4T1-PD1 cells also confirmed the overexpression of PD1 (Figure 2B). The genetically engineered 4T1-PD1 cells were then sonicated and centrifuged to obtain PD1 cell membranes, which were subsequently extruded through a pore-size polycarbonate membrane filter to produce PD1 NVs that still contained the modified PD1 protein (Figure 2C). The peak particle diameter of PD1 NVs was 163.1 ± 3.2 nm, and TEM images showed that the morphology was spherical vesicles (Figure 2D and S1A). Subsequently, Cur was loaded to PLGA nanoparticles by the microemulsion method to obtain Cur-PLGA nanoparticles, which had similar spherical morphology and particle size to PLGA, with a peak particle size of 149.4 ± 1.5 nm (Figure 2E and S1B). Furthermore, the DLC and DLE of Cur in Cur-PLGA were calculated based on the Cur standard curve, where the DLC of Cur in Cur-PLGA was 2.57% (Figure S2). Compared with Cur-NVs, the DLE of Cur in Cur-PLGA increased to 83.22%, indicating that using PLGA as a drug carrier can effectively improve the problem of insufficient loading efficiency of NVs (Figure S3).

To obtain PD1@Cur-PLGA, PD1 NVs were coated on the surface of Cur-PLGA. The core-shell structure observed by TEM proved that PD1@Cur-PLGA was successfully prepared. The particle size distribution of PD1@Cur-PLGA was 177 ± 5.7 nm (Figure 2F). Zeta potential revealed that PD1-Cur-PLGA had a surface charge similar to PD1 NVs, confirming that PD1 NVs were successfully coated onto Cur-PLGA (Figure 2G). In addition, the colocalization of DiO-labeled Cur-PLGA and DiI-labeled PD1 NVs observed in fluorescence images further demonstrated the successful fabrication of PD1@Cur-PLGA (Figure 2H). SDS-PAGE showed that PD1@Cur-PLGA and PD1 NVs had similar protein expression, indicating that PD1@Cur-PLGA retained membrane protein components after encapsulation (Figure 2I). The Cur absorption peak at 400-450 nm in the UV-vis absorption spectrum and the Cur spectroscopic properties at 450-650 nm in the fluorescence emission spectrum further proved that Cur-PLGA and PD1@Cur-PLGA were loaded with Cur (Figure 2J, K) 46. Cur-PLGA and PD1@Cur-PLGA greatly improved the solubility and stability of Cur (Figure S4), which remained stable in PBS and solutions containing 10% serum, with no significant changes in particle size and zeta potential over 7 days (Figure S5). The cumulative release of Cur from Cur-PLGA and PD1@Cur-PLGA was 59.638% and 58.315% at 84 h, respectively, and then reached a plateau (Figure S6). The release rate of Cur from Cur-PLGA was similar to that from PD1@Cur-PLGA, indicating that cell membrane coating did not affect the release of Cur. The above results indicated that PD1@Cur-PLGA was successfully prepared.

### *In vitro* cytotoxic effect of PD1@Cur-PLGA

**Figure 3:**
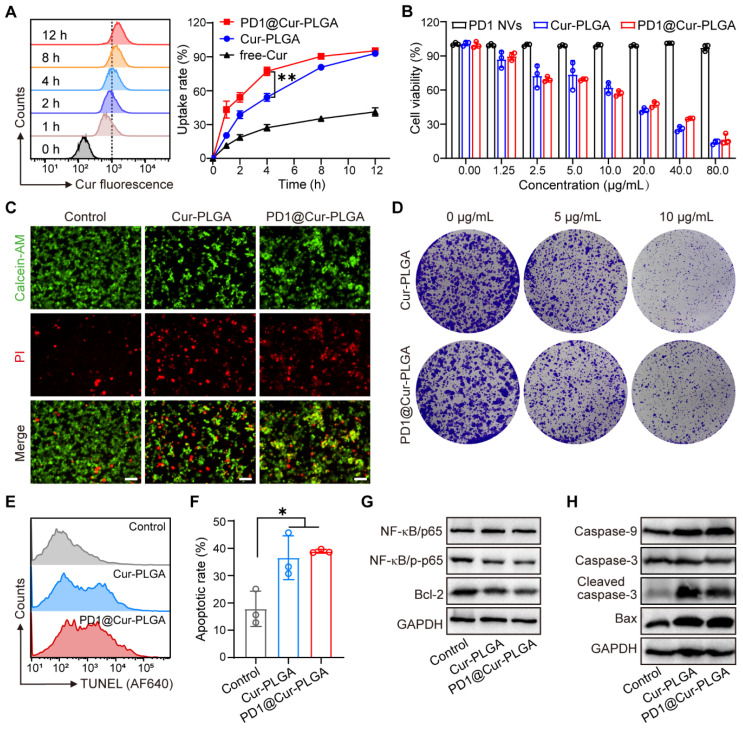
***In vitro* cellular uptake and cytotoxic effect of PD1@Cur-PLGA.** (A) Flow histograms of 4T1 cells after incubation with PD1@Cur-PLGA and quantitative analysis of uptake ratios of 4T1 cells after incubation with free-Cur, Cur-PLGA or PD1@Cur-PLGA (Cur, 20 μg/mL) at different times. (B) Cytotoxicity assay of 4T1 cells after different treatments. (C) Calcein-AM/PI fluorescence images of 4T1 cells after different treatments. Scale bar, 50 μm. (D) Representative photographs of 4T1 cell colonies after different treatments. (E, F) Representative histogram of TUNEL staining (E) and apoptosis rate (F) of 4T1 cells after various treatments. (G) WB analysis of NF-κB pathway and apoptosis inhibitory factor Bcl-2 in 4T1 cells after various treatments. (H) WB analysis of caspase-9, caspase-3, cleaved caspase-3, and Bax in 4T1 tumor cells after different treatments. All data are presented as mean values ± S.D. (*n* = 3). *P < 0.05, **P < 0.01.

Biomimetic membrane-coated nanomaterials have been widely studied due to their good biocompatibility and ability to prolong blood circulation time 47-49. After preparing PD1@Cur-PLGA, we first evaluated its blood compatibility through a hemolysis test. The results showed that hemolysis was minimal even at higher concentrations, indicating satisfactory compatibility (Figure S7). We then evaluated the cellular uptake in 4T1 cells through flow cytometry and CLSM. The uptake of PD1@Cur-PLGA was significantly higher than that of free-Cur and Cur-PLGA, indicating that PD1@Cur-PLGA has excellent tumor targeting due to the homologous targeting of PD1 NVs coated on the surface of the Cur-PLGA. (Figure 3A and S8). The internalization of Cur in 4T1 cells observed by CLSM images increased with prolonged incubation time, which also indicated the successful cellular uptake of PD1@Cur-PLGA (Figure S9).

After successfully demonstrating that PD1@Cur-PLGA could be effectively taken up by tumor cells, we further investigated its tumor-killing effect. The cytotoxic effect of PD1@Cur-PLGA was evaluated by CCK-8 assay, and the results demonstrated that the cytotoxicity of PD1@Cur-PLGA was Cur dose-dependent (Figure 3B). Furthermore, the cell viability of 4T1 cells after treatment with Cur-PLGA and PD1@Cur-PLGA was also characterized by live/dead cell staining assay. Cell death was observed when treated with Cur-PLGA and PD1@Cur-PLGA, indicating that they can significantly kill 4T1 cells (Figure 3C and S10A). A colony formation assay was performed to verify whether the cytotoxicity of Cur-PLGA and PD1@Cur-PLGA would affect the proliferation ability of 4T1 cells. Compared to the control group, the cell colonies in Cur-PLGA and PD1@Cur-PLGA groups were significantly reduced, indicating that both can inhibit the proliferation of 4T1 cells (Figure 3D and S10B). Next, the TUNEL assay was used to verify cell apoptosis after various treatments. Cur-PLGA and PD1@Cur-PLGA treatment induced obvious cell apoptosis and no difference in apoptosis rate, indicating that the cytotoxicity of Cur was not affected after cell membrane coating (Figure 3E, F). The NF-κB signaling pathway plays a vital role in regulating tumor immune responses, several studies have shown that NF-κB promotes tumor growth and leads to immune evasion by inducing the expression of anti-apoptotic proteins such as Bcl-2 50, 51. It has been reported that Cur can inhibit the NF-κB pathway and trigger apoptosis generation in tumor cells, thereby suppressing proliferation 25, 52. We next evaluated the effect of PD1@Cur-PLGA on the NF-κB signaling pathway by WB analysis. Compared with the untreated group, NF-κB/pho-p65 protein level was significantly downregulated after treatment with Cur-PLGA and PD1@Cur-PLGA. Furthermore, the expression of anti-apoptotic Bcl-2 protein was significantly decreased in 4T1 cells incubated with PD1@Cur-PLGA (Figure 3G). In contrast, protein levels of pro-apoptotic signals including caspase-9, cleaved caspase-3, and Bax were significantly increased (Figure 3H). These results indicated that PD1@Cur-PLGA can significantly inhibit cell proliferation and induce apoptosis.

### *In vitro* ICD effects and immune response activation of PD1@Cur-PLGA

**Figure 4:**
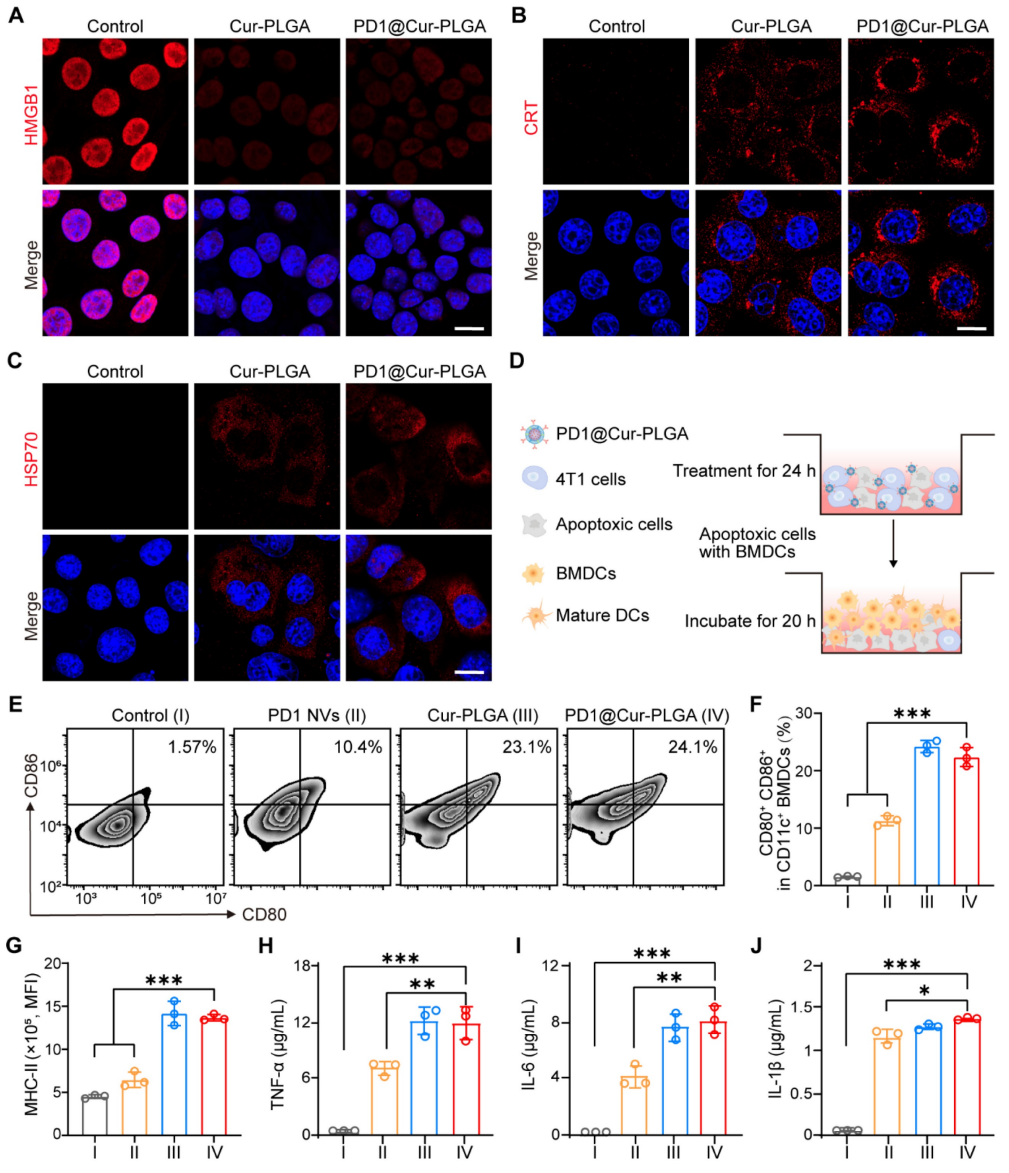
***In vitro* ICD effects and immune response activation of PD1@Cur-PLGA.** (A-C) Fluorescence images of HMGB1 (A), CRT (B), and HSP70 (C) were secreted in 4T1 cells after various treatments. Scale bar, 10 μm. (D) Schematic illustration of BMDCs co-cultured with apoptosis 4T1 cells* in vitro*. (E, F) Flow cytometry analysis (E) and quantitative analysis of mature DCs (CD80^+^CD86^+^) after various treatments. (G) Quantitative analysis of MHC-II^+^ (mean fluorescence intensity, MFI) in mature DCs. (H-J) Cytokine levels of TNF-α (H), IL-6 (I), and IL-1β (J) in the cell supernatants of BMDCs co-incubated with 4T1 apoptosis cells detected by ELISA. Control (I), PD1 NVs (II), Cur-PLGA (III), and PD1@Cur-PLGA (IV). Data are presented as mean values ± S.D. (*n* = 3). *P < 0.05, **P < 0.01, and ***P < 0.001.

ICD is regulated by the DAMPs released from dying tumor cells or expressed on the cell surface, mainly including HMGB1 and ATP secreted by tumor cells, as well as CRT and HSP70 exposed on the cell surface, which can induce DCs maturation and strengthen tumor immune responses 53, 54. PD1@Cur-PLGA-induced ICD was investigated by analyzing HMGB1 and ATP release, CRT exposure, and HSP70 expression in 4T1 cells. First, HMGB1 release was detected by ELISA and CLSM. Unlike control cells, where HMGB1 was mainly localized in the nucleus, an enhanced release of HMGB1 was observed in cells treated with Cur-PLGA and PD1@Cur-PLGA (Figure 4A). The ELISA results showed that the amount of HMGB1 released in the cell supernatants of the PD1@Cur-PLGA group was 2.1-fold higher than that of the untreated group (Figure S11A). Likewise, PD1@Cur-PLGA treatment significantly induced more CRT exposure and HSP70 expression (Figure 4B, C), and the mean fluorescence intensity of CRT and HSP70 in the Cur-PLGA and PD1@Cur-PLGA groups was approximately 19 times higher than that in the control group (Figure S11B). To detect the release of extracellular ATP, 4T1 cells were incubated with Cur-PLGA and PD1@Cur-PLGA for 24 h. PD1@Cur-PLGA treatment increased extracellular ATP secretion, and the relative ATP release rate was 4.87-fold that of the control group (Figure S11C). These results verified that PD1@Cur-PLGA could effectively induce the release of ICD-related DAMPs.

DAMPs can be recognized by DCs, the main type of antigen-presenting cells, leading to the upregulation of costimulatory molecules, thereby turning immature DCs into mature DCs 55, 56. To verify the effect of various treatments on DCs maturation, the 4T1 cancer cells pretreated with PD1 NVs, Cur-PLGA, and PD1@Cur-PLGA were co-cultured with BMDCs (Figure 4D and S12). The flow cytometry analysis showed that co-stimulatory molecules such as CD80, CD86, and MHC-II on the surface of BMDCs were significantly upregulated after various treatments (Figure 4E and S13). PD1 NVs promoted the maturation of BMDCs (CD80^+^CD86^+^) due to the natural antigen from cancer cell membranes (Figure 4F) 57. In addition, Cur-PLGA and PD1@Cur-PLGA-induced ICD also significantly promoted the maturation of BMDCs. Meanwhile, the expression level of MHC-II on BMDCs in the PD1@Cur-PLGA group was 2.55 times higher than that in the control group (Figure 4G). To verify the secretion levels of immune-related cytokines including tumor necrosis factor α (TNF-α), interleukin 6 (IL-6), and interleukin 1β (IL-1β), pretreated 4T1 cancer cells were incubated with BMDCs, and the supernatants were collected and analyzed by ELISA assay. The secretion level of TNF-α in PD1@Cur-PLGA groups was increased by 109 times compared to the control group (Figure 4H). The PD1@Cur-PLGA group exhibited the highest secretion of IL-6 and IL-1β, which are well-known pro-inflammatory factors (Figure 4I, J). These results indicated that PD1@Cur-PLGA can effectively promote the secretion of immune-related cytokines and enhance immune response.

### *In vitro* PDL1-specific binding and *in vivo* biodistribution of PD1@Cur-PLGA

**Figure 5:**
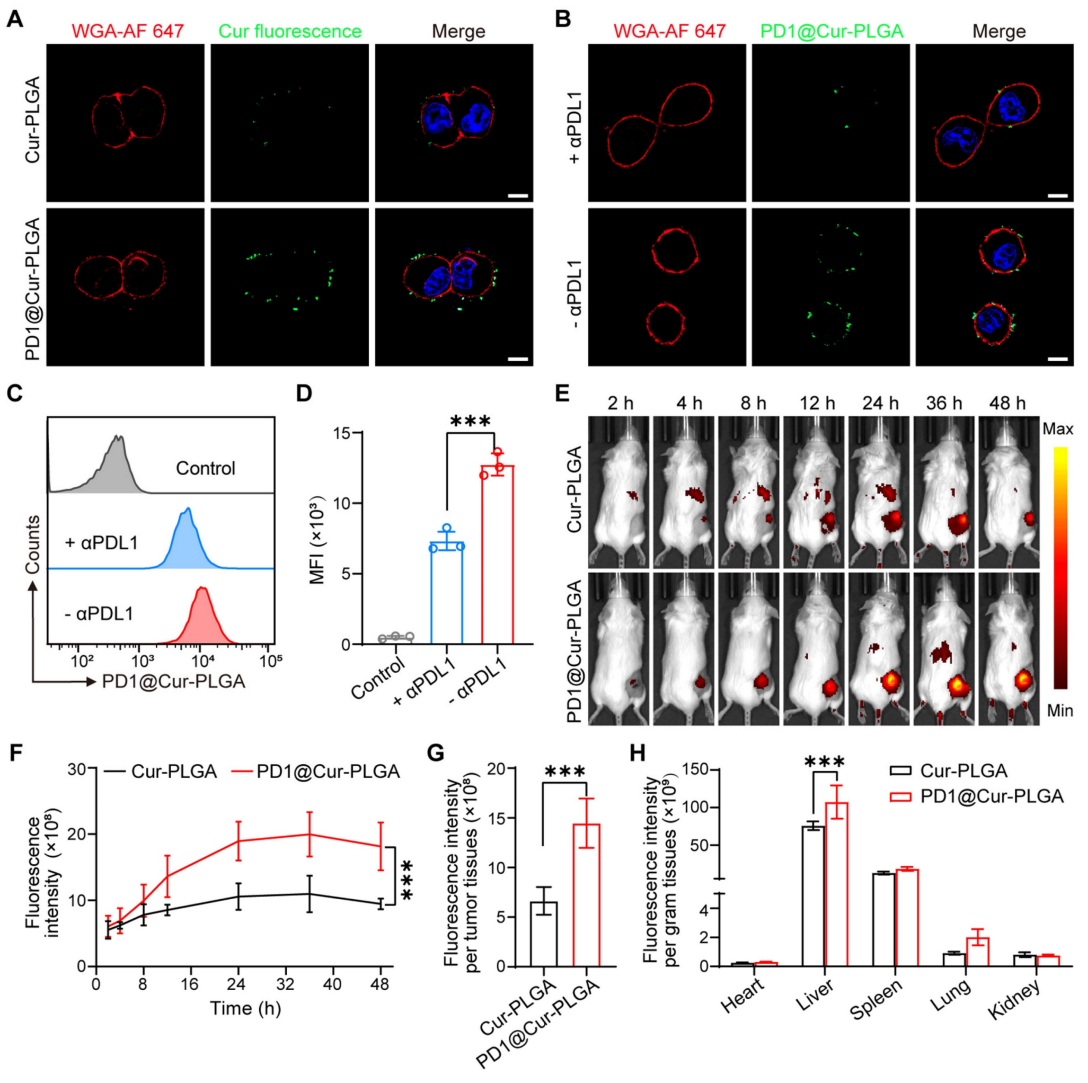
***In vitro* PDL1-specific binding and *in vivo* biodistribution of PD1@Cur-PLGA.** (A) Confocal fluorescence imaging of Cur-PLGA or PD1@Cur-PLGA binding to 4T1 cell membranes. Scale bar, 10 μm. (B) CLSM analysis of PD1@Cur-PLGA binding to 4T1 cell membranes. 4T1 cells were pretreated with or without *α*PD-L1 antibody before PD1@Cur-PLGA incubation. Scale bar, 10 µm. (C, D) Representative flow histograms (C) and quantitative analysis (D) of 4T1 cells after incubation with PD1@Cur-PLGA, the cells were pretreated with or without αPD-L1 antibody before PD1@Cur-PLGA incubation. (E, F) *In vivo* fluorescence imaging (E) and corresponding fluorescence intensity (F) of the mice at different time points after *i.v.* injection of an equivalent dose of DiR-labeled Cur-PLGA and PD1@Cur-PLGA. (G, H) The biodistribution of fluorescence in tumor tissues (G) and major organs (H). Data are shown as mean values ± S.D. (*n* = 3). ***P<0.001.

To investigate whether PD1@Cur-PLGA could target PDL1 on the surface of cancer cells, the binding of PD1@Cur-PLGA to 4T1 cell membranes was first assessed. Confocal fluorescence imaging demonstrated that PD1@Cur-PLGA bound to 4T1 cell membrane surface more effectively compared to Cur-PLGA (non-membrane-coated Cur-PLGA) and 4T1@Cur-PLGA (4T1 cell membrane-coated Cur-PLGA) (Figure 5A and S14A). Flow cytometry also showed that PD1@Cur-PLGA bound to 4T1 cells more than Cur-PLGA and 4T1@Cur-PLGA (Figure S14B, C). To further verify that the binding of PD1@Cur-PLGA to the 4T1 cell is the result of the interaction between PD1 on the surface of PD1@Cur-PLGA and PDL1 on the surface of the 4T1 cells, anti-PDL1 antibodies (αPDL1, 20 μg/mL) were added to 4T1 cells to pre-block PDL1 on the target cells 58. Compared with the PD1@Cur-PLGA group without αPDL1 pre-blocking, the binding rate of the αPDL1 + PD1@Cur-PLGA group was greatly reduced (Figure 5B). Flow cytometry analysis showed similar results, the fluorescence intensity of PD1@Cur-PLGA bound to 4T1 cells was significantly reduced after preincubation of αPDL1 with 4T1 cells (Figure 5C, D). These results indicated that the PD1 on the surface of PD1@Cur-PLGA could interact with tumor cells by binding between PD1/PDL1, which promotes targeting and infiltration at the tumor site and further exerts an effective antitumor effect.

To further investigate the systemic biodistribution of PD1@Cur-PLGA *in vivo*, DiR-labeled Cur-PLGA and DiR-labeled PD1@Cur-PLGA were intravenously injected into 4T1 tumor-bearing mice. The fluorescence signals were observed at the tumor site after PD1@Cur-PLGA injection for 4 h, then increased with time, and slightly weakened after 36 h (Figure 5E, F and S15A, B). Quantitative and statistical analysis of fluorescence signals showed that more PD1@Cur-PLGA accumulated in the tumor tissue compared with Cur-PLGA 48 h post-injection (Figure 5G and S15C). Major organs were also imaged 48 h post-injection, and the fluorescence of DiR-labeled PD1@Cur-PLGA was mainly in the liver and spleen (Figure 5H and S15D). Given that the nanoparticles partially accumulated in major organs, to evaluate the short-term toxicity of PD1@Cur-PLGA and its impact on major organ functions, major organs were harvested from mice 48 h after injection and analyzed. H&E-stained organ sections showed that compared with the PBS group, the heart, liver, spleen, kidney, and lung in the PD1@Cur-PLGA group had no significant damage (Figure S16A). Liver and renal function biomarkers remained within normal ranges, and these indices were comparable to those in the PBS group (Figure S16B). These results indicated that PD1@Cur-PLGA had excellent tumor targeting and favorable biosafety.

### *In vivo* antitumor effect of PD1@Cur-PLGA

**Figure 6:**
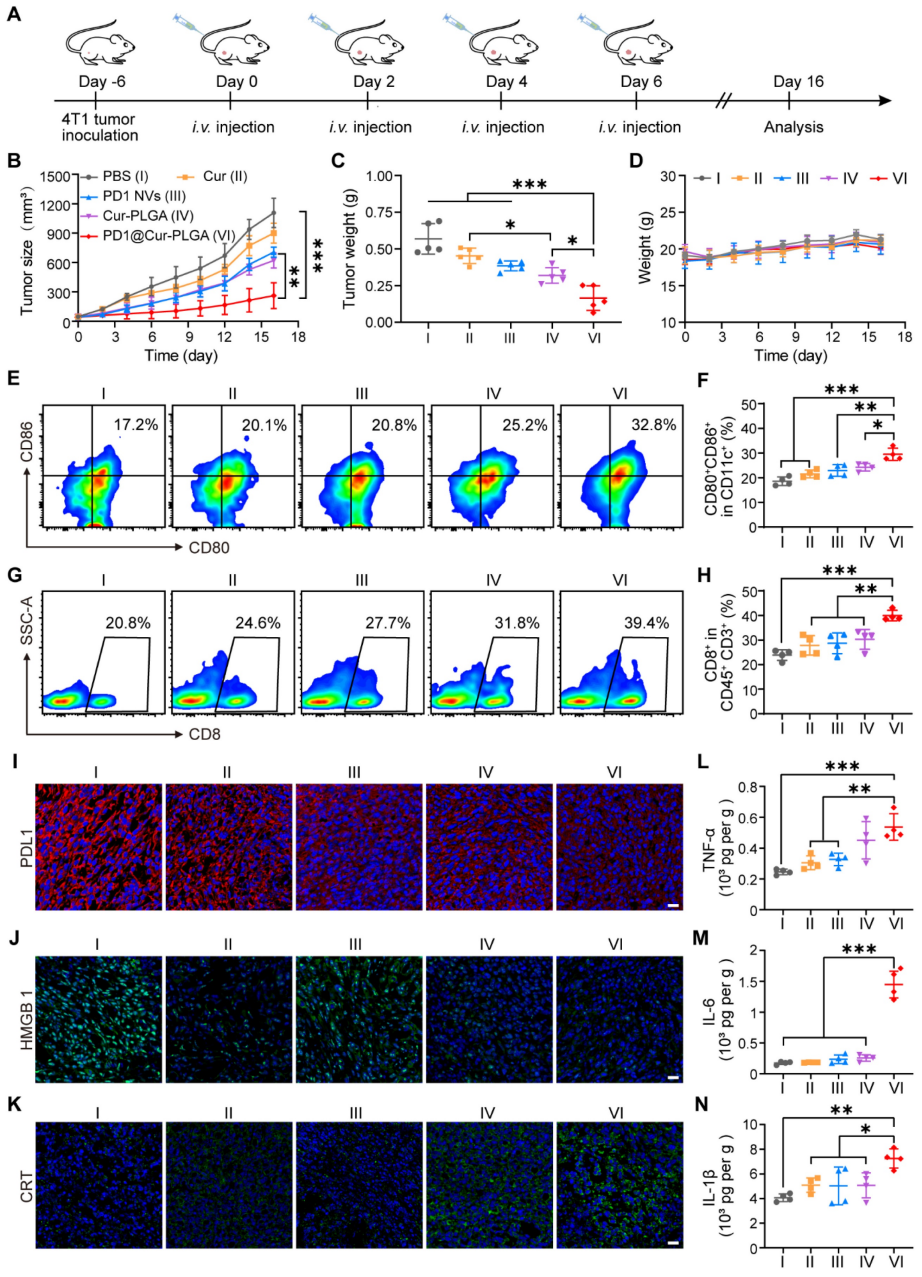
***In vivo* antitumor effect and immune response activation of PD1@Cur-PLGA.** (A) Experimental timeline for treatment efficacy studies. (B) Growth profiles of 4T1 tumors in mice receiving the indicated treatments (*n* = 5). (C) Weights of 4T1 tumors following different treatments. (D) Change in mice body weights during various treatments. (E-H) Representative of flow cytometry plots and quantification of the proportions of (E, F) matured DCs (CD80^+^CD86^+^ gated on CD11c^+^ cells) and (G, H) CD8^+^ T cells (gated on CD45^+^CD3^+^ cells) in tumor tissues after injections of various nanoparticles (*n* = 4). (I-K) Fluorescence staining analysis of PDL1 levels (I), CRT expression (J), and HMGB1 release level (K) in tumors after various treatments. Scale bar, 20 μm. (L-N) ELISA analysis of TNF-α (L), IL-6 (M), IL-1β (N) in tumor tissues after various treatments (*n* = 4). Data are shown as mean values ± S.D. *P<0.05, **P<0.01, and ***P<0.001.

To evaluate the inhibition effects of different nanoparticles on tumor growth, 4T1 tumor-bearing mice were injected with nanoparticles *via* the tail vein at two-day intervals (Figure 6A). As shown in Figure 6B, tumors in the PBS group grew rapidly, while the Cur group only showed a minor effect on tumor growth due to its low bioavailability in the body. In contrast, the PD1 NVs and Cur-PLGA groups had moderate inhibitory effects on tumor growth because Cur-PLGA can induce ICD to attenuate the immunosuppressive environment in solid tumors, and PD1 NVs can specifically bind to PDL1 on the tumor surface to enhance ICB. It is worth noting that PD1@Cur-PLGA combined the advantages of Cur-PLGA and PD1 NVs, effectively suppressed tumor growth (Figure S17). At the end of the treatment, the PD1@Cur-PLGA group had the smallest tumor weight, thanks to its significant tumor-targeting ability and excellent overall therapeutic effects (Figure 6C). Moreover, the treated mice revealed no significant injury to major organs, no change in liver and kidney function indices in their serum, and no weight loss (Figure 6D and S18). These results indicated that PD1@Cur-PLGA combined the advantages of Cur-PLGA and PD1 NVs for antitumor therapy, exhibiting significant tumor growth inhibition and excellent biocompatibility.

To further evaluate the treatment efficacy of PD1@Cur-PLGA against other tumors, a prostate tumor model was established by subcutaneous injection of RM-1 cells into C57BL/6 mice. When the tumor volume reached 50-100 mm^3^, it was set as day 0, and then different treatments were administered on days 0, 2, 4, and 6, respectively (Figure S19A). The tumor volume of RM-1 tumor-bearing mice treated with PD1@Cur-PLGA was smaller than that in the PBS group (Figure S19B, C), further demonstrating that PD1@Cur-PLGA has a significant antitumor therapeutic effect. Moreover, the PD1@Cur-PLGA group had a lighter tumor weight at day 10, showing a good therapeutic effect (Figure S19D). The body weight of mice in the PD1@Cur-PLGA group were similar to that of PBS group throughout the whole treatment (Figure S19E), indicating that PD1@Cur-PLGA has few side effects and good biocompatibility. To better understand the mechanism of PD1@Cur-PLGA mediated immunotherapeutic efficacy, we assessed the pathological status of RM-1 prostate tumor tissues at day 10 after various treatments by H&E, Ki67 staining, and CD8 immunofluorescence staining assay. As shown in Figure S19F, H&E staining of tumor slices revealed that most tumor cells in the PD1@Cur-PLGA group had obvious nuclear atrophy, cellular destruction, and tumor necrosis when compared with the PBS group. Similarly, the expression of Ki67 was decreased after PD1@Cur-PLGA treatment, indicating that PD1@Cur-PLGA-mediated immunotherapy effectively suppressed tumor growth by inhibiting tumor proliferation and inducing apoptosis. The PD1@Cur-PLGA group had more CD8^+^ T cell infiltration. In summary, PD1@Cur-PLGA combines the advantages of Cu-PLGA and PD1 NVs, inhibiting tumor growth while also initiating specific PD1/PDL1 axis-mediated immune responses, thereby producing synergistic immunotherapy.

### Antitumor immune response of PD1@Cur-PLGA

Tumor-infiltrating immune cells were further analyzed by flow cytometry to explore the tumor growth inhibition mechanism of PD1@Cur-PLGA. DCs are a heterogeneous group of antigen-presenting cells that are crucial for both innate and adaptive immune response initiation and regulation 59, 60. The release of tumor-associated antigens induces the maturation of DCs in draining lymph nodes, which promotes its antigens presenting to initiate the activation and recruitment of T cells and tumor-infiltrating T cells to induce adaptive antitumor immunity 61, 62. The representative diagram of flow cytometric gating/sorting strategies is shown in Figure S20. The percentage of matured DCs in the PD1@Cur-PLGA group was 1.77-, 1.53-, 1.44-, and 1.35-folds higher than that of the PBS, Cur, PD1 NVs, and Cur-PLGA groups, respectively (Figure 6E, F), indicating its great potential in eliciting strong body immune response. CD8^+^ T cells are one of the main members of CTLs, and the proportion of CD8^+^ T cells is closely related to the efficiency of the anticancer immune response 63, 64. The PD1@Cur-PLGA group significantly increased the tumor infiltration of CD8^+^ T cells, and the percentages of CD8^+^ T cells were 1.6-, 1.4-, 1.34-, and 1.25 times higher than those of the PBS, Cur, PD1 NVs, and Cur-PLGA groups, respectively (Figure 6G, H). These results suggested that PD1@Cur-PLGA-mediated treatment can stimulate humoral immune responses, thereby promoting immune cell infiltration and attack on tumor cells.

Meanwhile, immunofluorescence analysis showed that PD1@Cur-PLGA treatment significantly reduced PDL1 expression in tumor tissues (Figure 6I). Moreover, compared to the PBS group, the immunofluorescence signal of CRT in the PD1@Cur-PLGA group was significantly enhanced, while the immunofluorescence signal of HMGB1 was reduced, verifying the induction of the ICD effect *in vivo* (Figure 6J, K). The PD1-mediated ICB and Cur-induced ICD combination enables PD1@Cur-PLGA to exhibit the best antitumor effect. Moreover, PD1@Cur-PLGA significantly increased the pro-inflammatory factors TNF-α, IL-6, and IL-1β in tumor tissues (Figure 6L-N), indicating the successful elicitation of robust antitumor immune responses. These results suggested that PD1@Cur-PLGA effectively strengthens antitumor immune responses, thereby enhancing the tumor inhibition effect.

## Conclusion

In conclusion, we have successfully developed genetically engineered NVs-coated PD1@Cur-PLGA nanoparticles that integrate ICD and ICB therapy to enhance tumor immunotherapy. The genetically engineered NVs greatly improved tumor targeting and accumulation of the nanoparticles, and disruption of the PD1/PDL1 signaling pathway by PD1 greatly enhanced cancer immunotherapy by promoting T cell infiltration and antitumor immune responses. The PLGA significantly improved the hydrolytic stability and bioavailability of Cur, and the released Cur successfully induced tumor cell apoptosis by inhibiting NF-κB phosphorylation and Bcl-2 protein expression as well as activating caspase and Bax apoptotic signaling, which triggered effective ICD to elicit the immune response. Combining the immunostimulatory effects of ICD and ICB, PD1@Cur-PLGA exhibited potent immune responses and tumor growth inhibition in a 4T1 breast tumor-bearing mouse model. These encouraging results highlight the advantages and broad perspectives of genetically engineered NVs in enhancing the synergistic therapy of ICD and ICB, providing a more meaningful reference for the development of low-cost, safe, and robust tumor immunotherapy.

## Supplementary Material

Supplementary figures.
