# Supplementary material for: Genetically engineered cellular nanoparticles loaded with curcuminoids for cancer immunotherapy
Source: Theranostics. 2024 Oct 7;14(16):6409–25. doi: 10.7150/thno.99033 (PMC11488111; doi:10.7150/thno.99033)
Supplement: Supplementary file 1 — Supplementary figures. [file thnov14p6409s1.pdf]

Supplementary Materials

Genetically engineered cellular nanoparticles loaded with curcuminoids for cancer immunotherapy

Yifang Liao[1,3,#], Chenchen Zhao[2,#], Yuanwei Pan[2,#], Yiming Guo[1,2], Lujie Liu[2], Jicheng Wu[2,4], Yunjiao Zhang[3,*], Lang Rao[2,*], and Qi Li[1,*]

[1] Department of Urology, The First Affiliated Hospital of Zhengzhou University, Zhengzhou 450052, China.

[2] Institute of Chemical Biology, Shenzhen Bay Laboratory, Shenzhen 518132, China.

[3] School of Medicine, South China University of Technology, Guangzhou 510006, China.

[4] Cancer Center, Renmin Hospital of Wuhan University, Wuhan 430060, China.

[#] These authors contributed equally to this work.

* Corresponding authors: zhangyunjiao@scut.edu.cn (Y.Z.); lrao@szbl.ac.cn (L.R.); richee@zzu.edu.cn (Q.L.).

Supplementary Figures

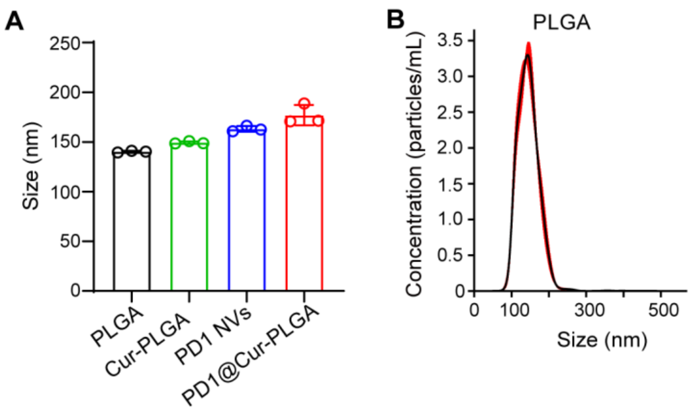

Figure S1. (A) Hydrodynamic size of PLGA, Cur-PLGA, PD1 NVs, and PD1@Cur-PLGA. (B) Size distribution of PLGA. All data are shown as mean ± S.D. (n = 3).

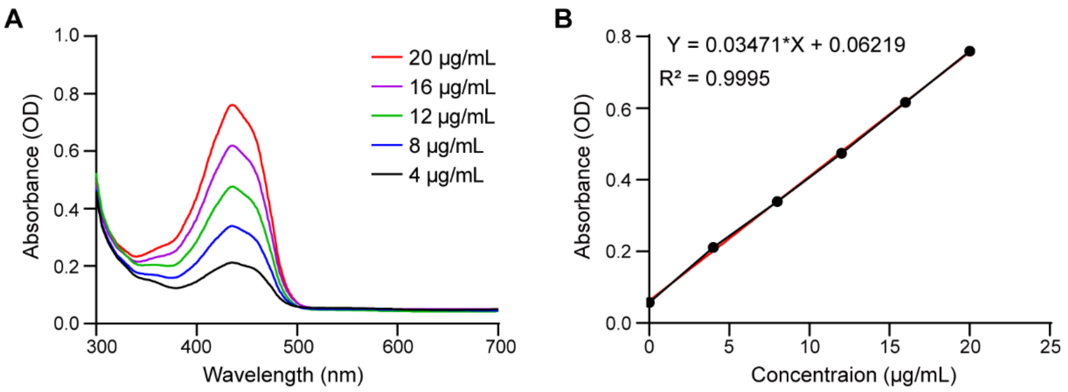

Figure S2. (A) UV-vis spectra Cur solutions with different concentrations. (B) Standard curve of Cur solution calculated using absorbance at 435 nm.

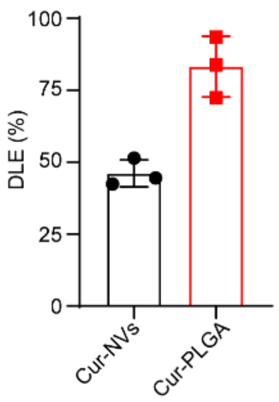

Figure S3. Drug loading efficiency (DLE) of Cur in NVs and PLGA. All data are shown as mean ± S.D. (n = 3).

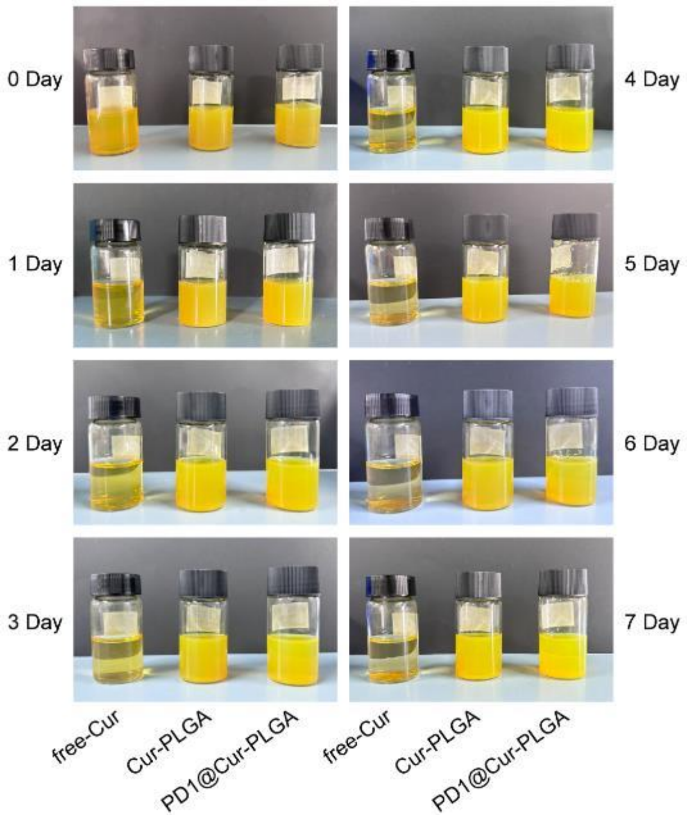

Figure S4. Photograph of free-Cur, Cur-PLGA, and PD1@Cur-PLGA solutions in PBS at different times.

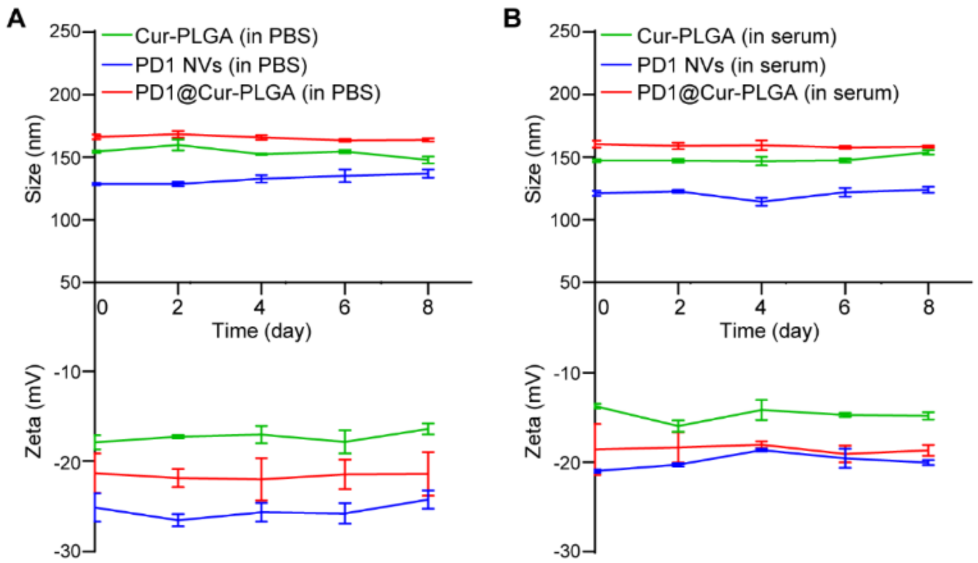

Figure S5. Stability of Cur-PLGA, PD1 NVs, and PD1@Cur-PLGA solutions in PBS (A) or serum (B) over 7 days. All data are shown as mean ± S.D. (n = 3).

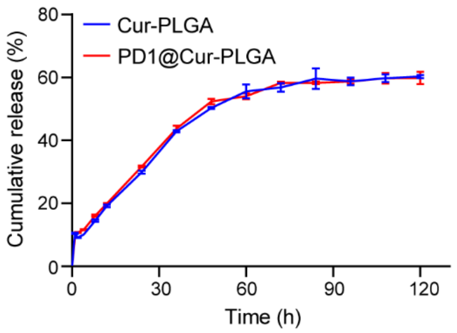

Figure S6. *In vitro* release profiles of Cur from Cur-PLGA or PD1@Cur-PLGA. All data are shown as mean ± S.D. (n = 3).

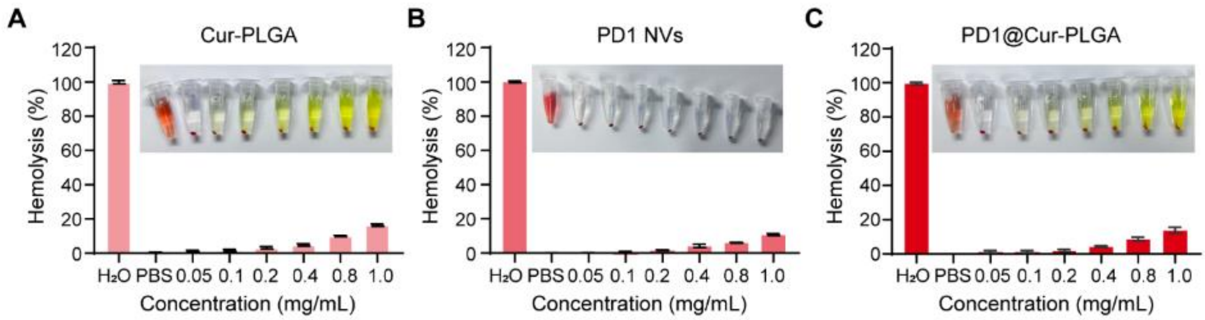

Figure S7. Hemolysis test of Cur-PLGA, PD1 NVs, and PD1@Cur-PLGA with different concentrations. All data are shown as mean ± S.D. (n = 3).

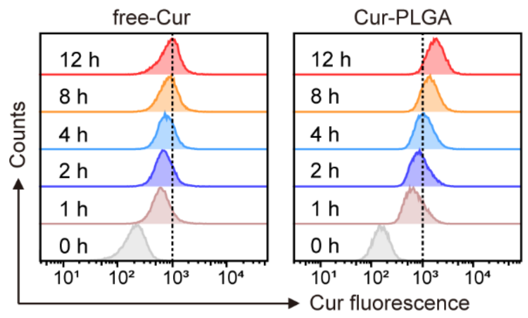

Figure S8. Representative histograms of uptake ratios of 4T1 cells after incubation with free-Cur or Cur-PLGA (Cur, 20 μg/mL) at different times. Data are presented as mean values ± S.D. (*n* = 3).

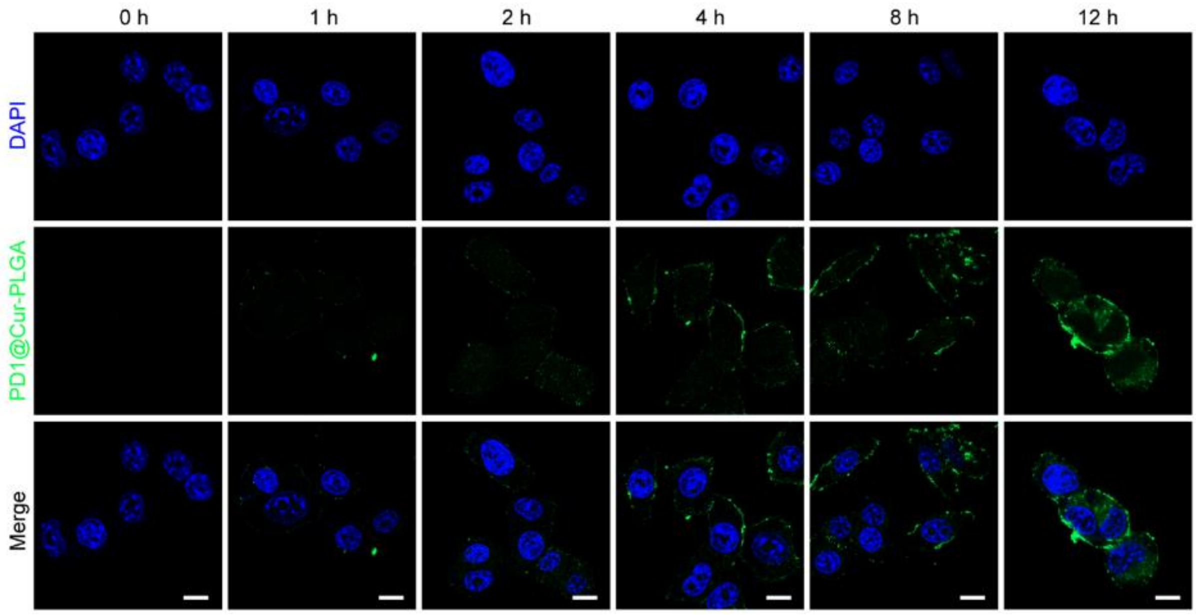

Figure S9. Cellular uptake of PD1@Cur-PLGA in 4T1 cells after different incubation times. Scale bar, 10 μm.

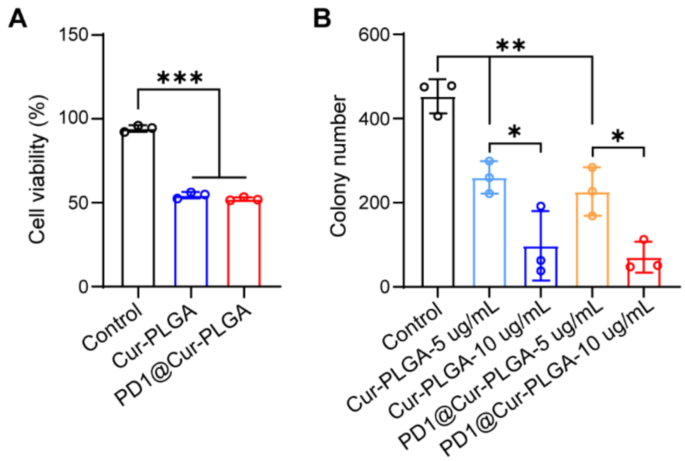

Figure S10. Statistical analysis of live/dead cell staining (Calcein-AM/PI) assay (A) and colony formation assay (B). All data are shown as mean ± S.D. (n = 3). *$P < 0.05$, **$P < 0.01$, ***$P < 0.001$.

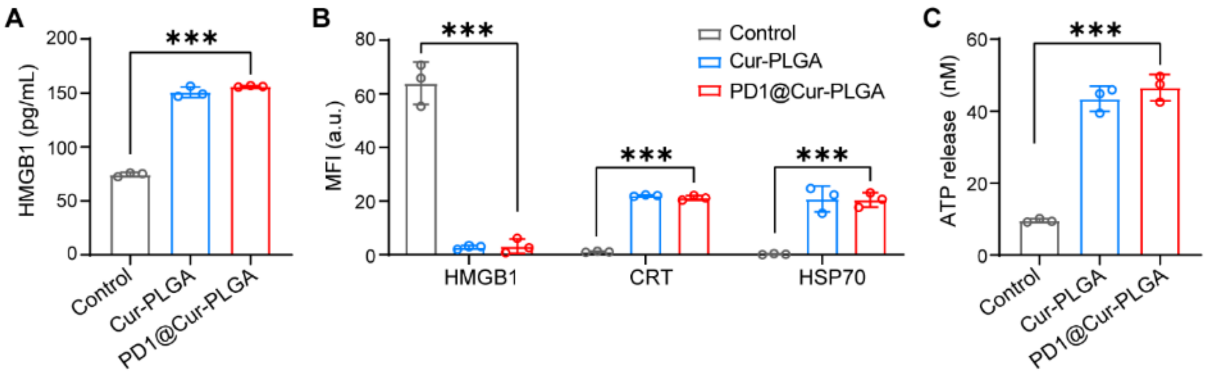

Figure S11. *In vitro* ICD effect triggered by PD1@Cur-PLGA. (A) ELISA analysis of HMGB1 released by 4T1 cells after different treatments for 24 h. (B) Statistical analysis of HMGB1, CRT, and HSP70 expression after different treatments. (C) Statistical analysis of extracellular ATP levels in 4T1 cells after different treatments. All data are shown as mean ± S.D. ($n = 3$). ***$P < 0.001$.

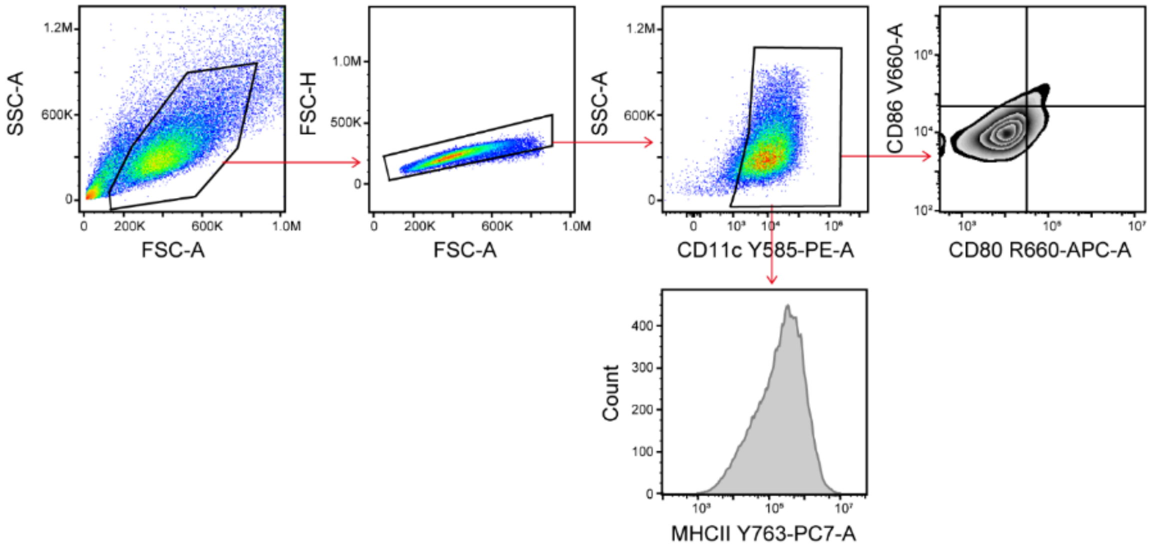

Figure S12. The gating strategy of CD80^{+}CD86^{+} and MHC-II in BMDCs in Figure 4E and Figure S13.

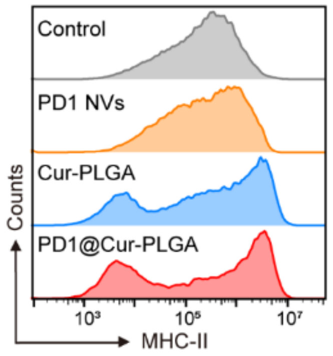

Figure S13. Representative histograms of MHC-II expression in BMDCs after different treatments.

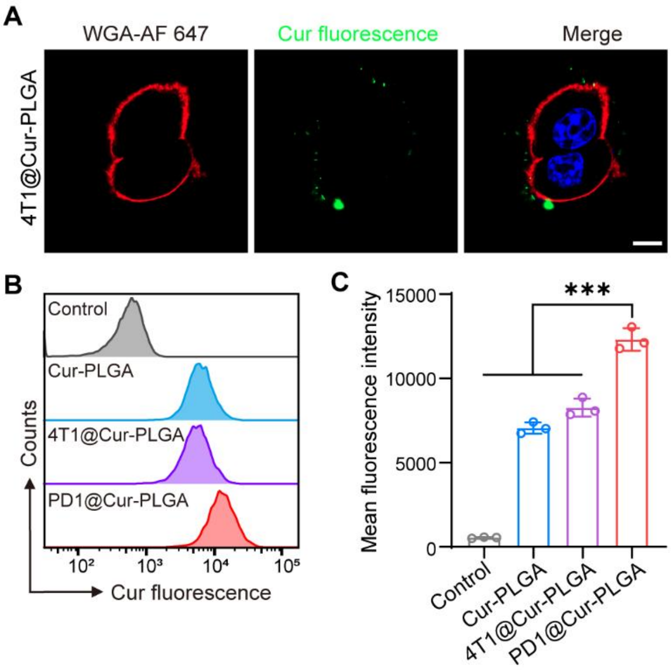

Figure S14. The targeting effect of PD1@Cur-PLGA to tumor cells. (A) Fluorescence imaging analysis of 4T1@Cur-PLGA binding to 4T1 cell membrane. Scale bar, 10 μm. (B, C) Flow cytometry histograms (B) and mean fluorescence intensity quantitative analysis (C) of 4T1 cells after incubation with different nanoparticles. Data are represented as mean values ± S.D. (n = 3). ***P < 0.001.

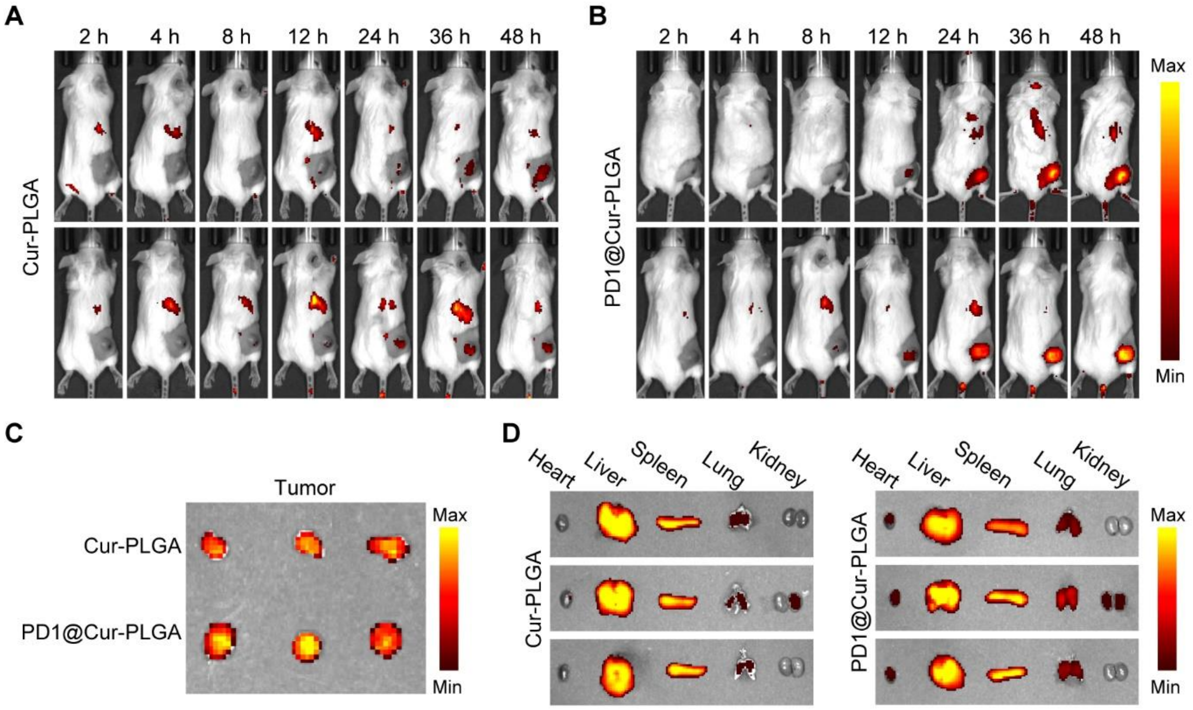

Figure S15. *In vivo* biodistribution of PD1@Cur-PLGA. (A, B) *In vivo* fluorescence imaging of the mice at different time points after *i.v.* injection of an equivalent dose of DiR-labeled Cur-PLGA (A) and PD1@Cur-PLGA (B). (C, D) Biodistribution of Cur-PLGA and PD1@Cur-PLGA in tumor tissues (C) and major organs (D) after 48 h of injection.

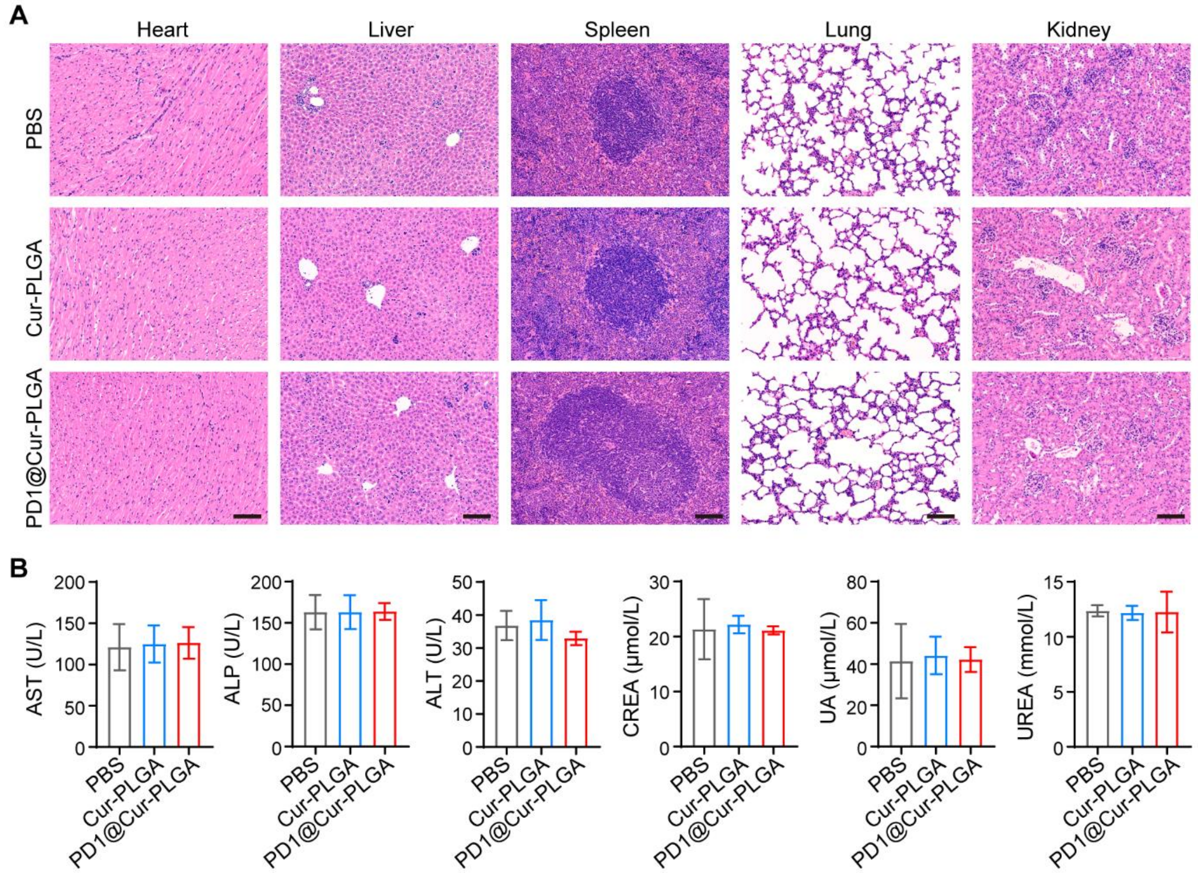

Figure S16. Toxicity evaluation of PD1@Cur-PLGA. (A) Representative pathological H&E staining of major organs after PBS, Cur-PLGA, or PD1@Cur-PLGA treatments for 48 h. Scale bar, 100 μm. (B) Analysis of serum biochemistry markers for liver (AST, ALP, and ALT) and kidney (CREA, UA, and UREA) after different treatments for 48 h. Data are represented as mean values ± S.D. (*n* = 3). (AST: aspartate aminotransferase; ALP: alkaline phosphatase; ALT: alanine transaminase; CREA: creatinine; UA: uric acid; UREA: urea)

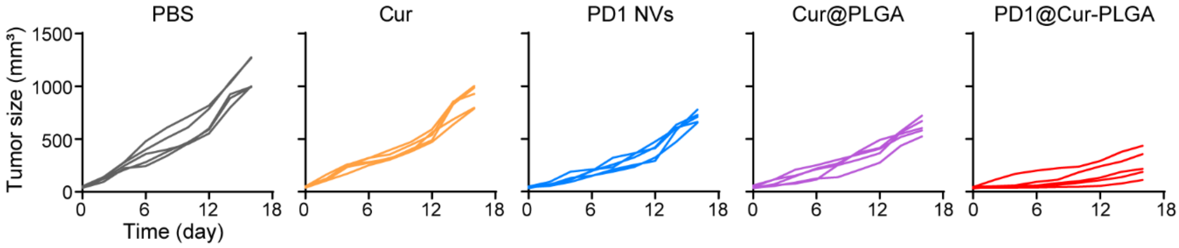

Figure S17. Individual 4T1 tumor growth curves after treatment with PBS, Cur, PD1 NVs, Cur-PLGA, and PD1@Cur-PLGA ($n = 5$).

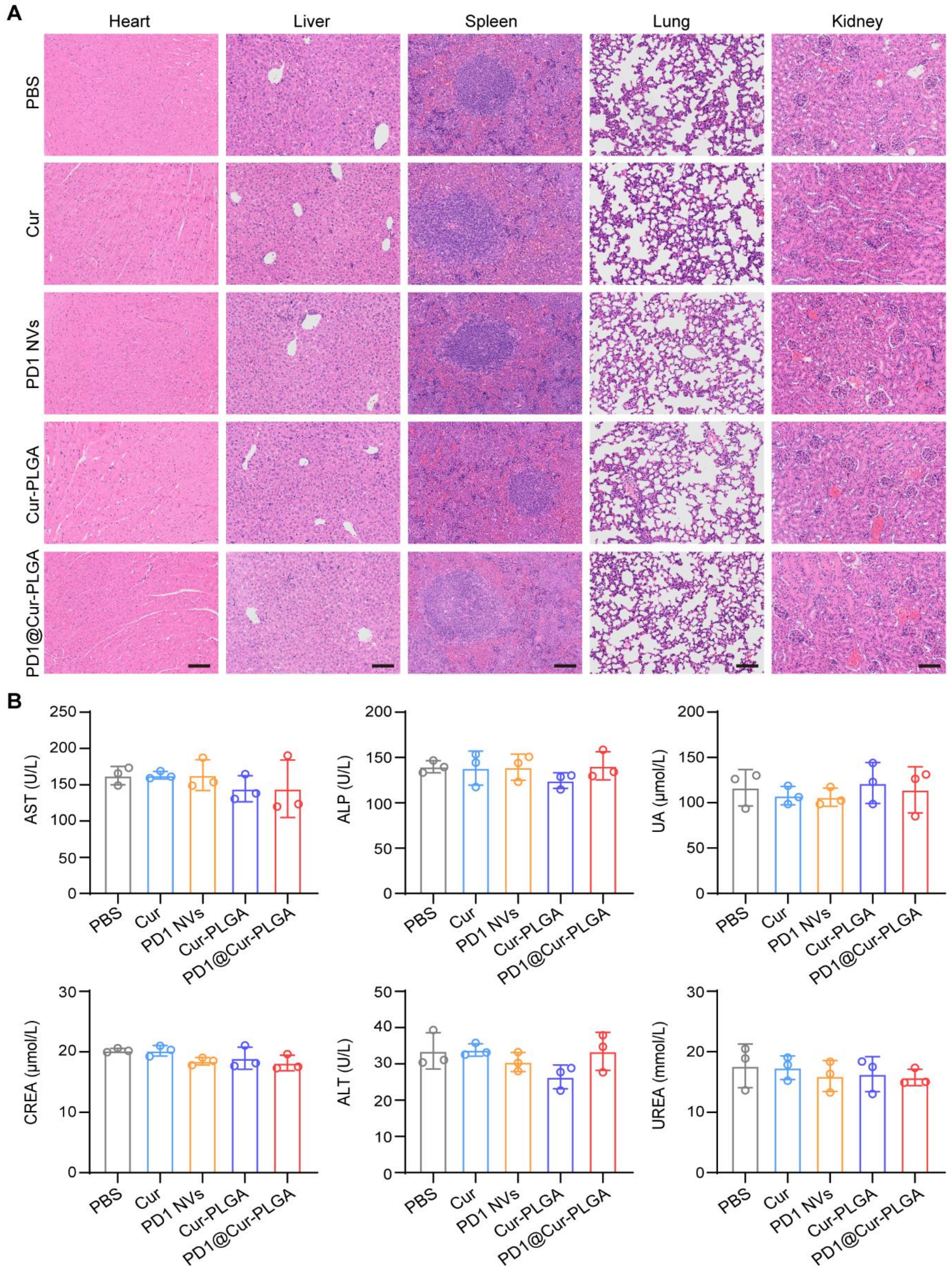

Figure S18. Biosafety evaluation of different nanoparticles. (A) Representative pathological H&E-stained images of major organs and (B) blood biochemistry analysis of the mice after various treatments. Scale bar, 100 μm. The results were expressed as the mean ± S.D. (*n* = 3).

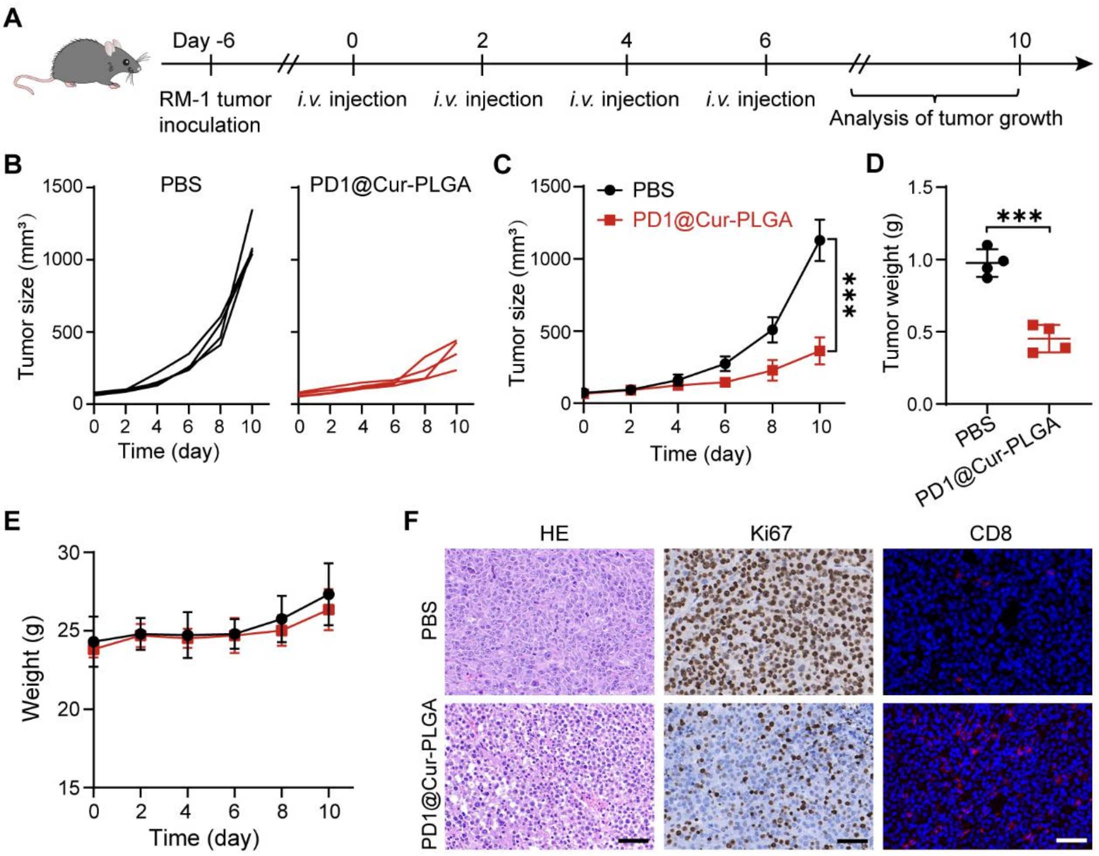

Figure S19. Antitumor efficacy of PD1@Cur-PLGA *in vivo*. (A) Schematic representation of therapy timeline in the C57BL/6 mice RM-1 prostate tumor model. (B) Individual RM-1 prostate tumor growth curves after treatment with PBS and PD1@Cur-PLGA. (C) Growth profiles of RM-1 prostate tumors in C57BL/6 mice receiving the indicated treatments. (D) Weights of RM-1 prostate tumors at the end of different therapy. (E) Change in mice body weights during various treatments. (F) Immunohistochemical analysis of HE, Ki-67, and immunofluorescence analysis of CD8 in RM-1 prostate tumor after different therapy. Scale bar, 50 μm. All data are shown as mean values ± S.D. ($n = 4$). $***P < 0.001$.

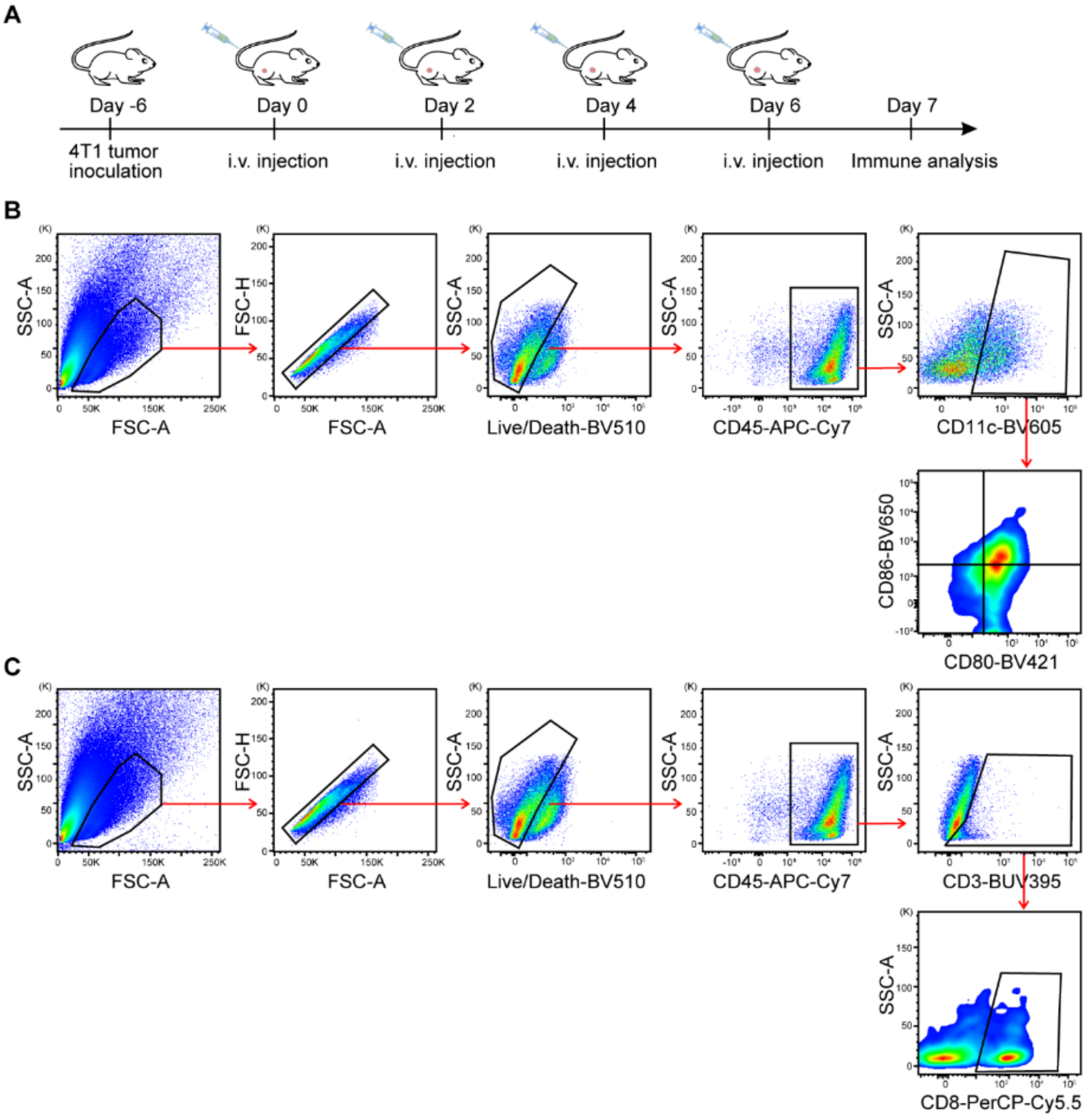

Figure S20. Scheme of *in vivo* antitumor immunity assessment and flow cytometry gating strategy to analyze different infiltrating immune cells tumor tissue. (A) Schematic diagram of evaluating anti-tumor immune effects *in vivo*. (B) The gating strategy of $CD80^+CD86^+$ DCs in Figure 6G. (C) The gating strategy of $CD8^+$ T cells in Figure 6E.
